# Oncogenic plasmid DNA and liver injury agent dictates liver cancer development in a mouse model

**DOI:** 10.1042/CS20240560

**Published:** 2024-09-26

**Authors:** Vincent Chiu, Christine Yee, Nathan Main, Igor Stevanovski, Matthew Watt, Trevor Wilson, Peter Angus, Tara Roberts, Nicholas Shackel, Chandana Herath

**Affiliations:** 1 Ingham Institute for Applied Medical Research, Liverpool, New South Wales, Australia; 2 South Western Sydney Clinical School, UNSW Sydney, Liverpool, New South Wales, Australia; 3 School of Biomedical Sciences, University of Melbourne, Victoria, Australia; 4 Hudson Institute of Medical Research, Monash University, Victoria, Australia; 5 Department of Gastroenterology and Hepatology, Austin Health, Heidelberg, Victoria, Australia; 6 School of Medicine, Western Sydney University, Campbelltown, New South Wales, Australia; 7 Department of Medicine, Austin Health, University of Melbourne, Victoria, Australia

**Keywords:** hepatic steatosis, hydrodynamic tail vein injection, Liver cancer, liver fibrosis, liver injury, thioacetamide

## Abstract

Primary liver cancer is an increasing problem worldwide and is associated with significant mortality. A popular method of modeling liver cancer in mice is plasmid hydrodynamic tail vein injection (HTVI). However, plasmid-HTVI models rarely recapitulate the chronic liver injury which precedes the development of most human liver cancer. We sought to investigate how liver injury using thioacetamide contributes to the pathogenesis and progression of liver cancer in two oncogenic plasmid-HTVI-induced mouse liver cancer models. Fourteen-week-old male mice received double-oncogene plasmid-HTVI (SB/AKT/c-Met and SB/AKT/NRas) and then twice-weekly intraperitoneal injections of thioacetamide for 6 weeks. Liver tissue was examined for histopathological changes, including fibrosis and steatosis. Further characterization of fibrosis and inflammation was performed with immunostaining and real-time quantitative PCR. RNA sequencing with pathway analysis was used to explore novel pathways altered in the cancer models. Hepatocellular and cholangiocellular tumors were observed in mice injected with double-oncogene plasmid-HTVI models (SB/AKT/c-Met and SB/AKT/NRas). Thioacetamide induced mild fibrosis and increased alpha smooth muscle actin-expressing cells. However, the combination of plasmids and thioacetamide did not significantly increase tumor size, but increased multiplicity of small neoplastic lesions. Cancer and/or liver injury up-regulated profibrotic and proinflammatory genes while metabolic pathway genes were mostly down-regulated. We conclude that the liver injury microenvironment can interact with liver cancer and alter its presentation. However, the effects on cancer development vary depending on the genetic drivers with differing active oncogenic pathways. Therefore, the choice of plasmid-HTVI model and injury agent may influence the extent to which injury promotes liver cancer development.

## Introduction

Primary liver cancer is the sixth most common cancer worldwide and the third leading cause of cancer death [1]. The majority of primary liver cancer cases are hepatocellular carcinoma (HCC) followed by intrahepatic cholangiocarcinoma [2,3], which have poor long-term survival in patients not eligible for surgical resection [4,5]. Common causes of HCC include chronic infection with hepatitis viruses B and C, Metabolic dysfunction-associated steatotic liver disease/metabolic dysfunction-associated steatohepatitis (MASLD/MASH) and chronic alcohol consumption [5]. While many cases of intrahepatic cholangiocarcinoma are sporadic, risk factors include cholestatic diseases, chronic liver infections and liver cirrhosis [6]. Incidence and mortality from liver cancers have dramatically increased over the past several decades [2,7,8]. Thus, there is need for greater understanding of the processes of disease progression to combat this growing health problem.

An emergent method of modelling liver cancer in mice is hydrodynamic tail vein injection (HTVI). HTVI of oncogene expression plasmids together with a transposase plasmid such as Sleeping Beauty (SB) results in genomic integration of constitutively-expressed oncogenes primarily within the liver [9–11]. This can be used to generate a liver cancer model with a well-defined gain-of-function without the need to generate and maintain transgenic mouse lines. The modular nature of plasmid-HTVI allows many combinations of oncogenes and mouse strains/genetic backgrounds to be developed rapidly [12]. Furthermore, plasmid-HTVI models have the potential to be faster than conventional carcinogen models, with some plasmid combinations developing tumors in as little as four weeks [11,13–15].

Regardless of etiology, common pathogenic drivers of liver cancer in humans are inflammation and fibrosis resulting from chronic injury [16,17]. Thus, the cancer-promoting property of chronic liver injury has been used to increase the tumorigenicity of mouse models of liver cancer. Hepatotoxins such as carbon tetrachloride (CCl_4_) [18,19] and thioacetamide (TAA) [20,21], and dietary injury such as high-fat diet [20,22], methionine-choline-deficient diet [23] and alcohol [24] have been employed as liver injury agents in animal models.

However, a limitation of most plasmid-HTVI studies is that they do not model development of liver cancer on a background of liver injury and fibrosis, which is the context of most human liver cancers [12]. Without liver injury, these models do not reflect the microenvironment in which tumors develop. Combining plasmid-HTVI models with liver injury can therefore be used to investigate how liver injury modulates the progression of carcinogenesis and cancer phenotypes by providing the relevant microenvironment. However, there are relatively few studies combining plasmid-HTVI models with liver injury agents compared with those without liver injury agents [25–29]. Given the large number of possible plasmid-injury combinations, it is unknown whether the interactions between different plasmid-HTVI models and liver injury agents uniformly promote cancer progression. The Ras-mitogen activated protein kinase and phosphatidylinositol-3-kinase (PI3K)-AKT-mammalian target of rapamycin (mTOR) pathways are known to be activated in HCC and *MET* is known to be overexpressed in HCC [30]. Therefore, the present study aimed to characterize plasmid-HTVI models overexpressing active forms of AKT, c-Met and NRas combined with TAA as a liver injury agent to elucidate pathogenic pathways associated with cancer progression in these models.

## Methods

### Animal experiments

All animal experiments were performed in accordance with the Australian Code for the Care and use of Animals for Scientific Purposes and ARRIVE guidelines with approval from the Animal Care and Ethics Committee of UNSW Sydney (approval number 18/23A). Experiments were performed at the Ingham Institute for Applied Medical Research. Animals were housed in specific pathogen-free conditions on a 12-h light/dark cycle with *ad libitum* access to chow (Gordon’s Specialty Stock Feeds, NSW, Australia) and acidified reverse osmosis water.

**Figure 1:**
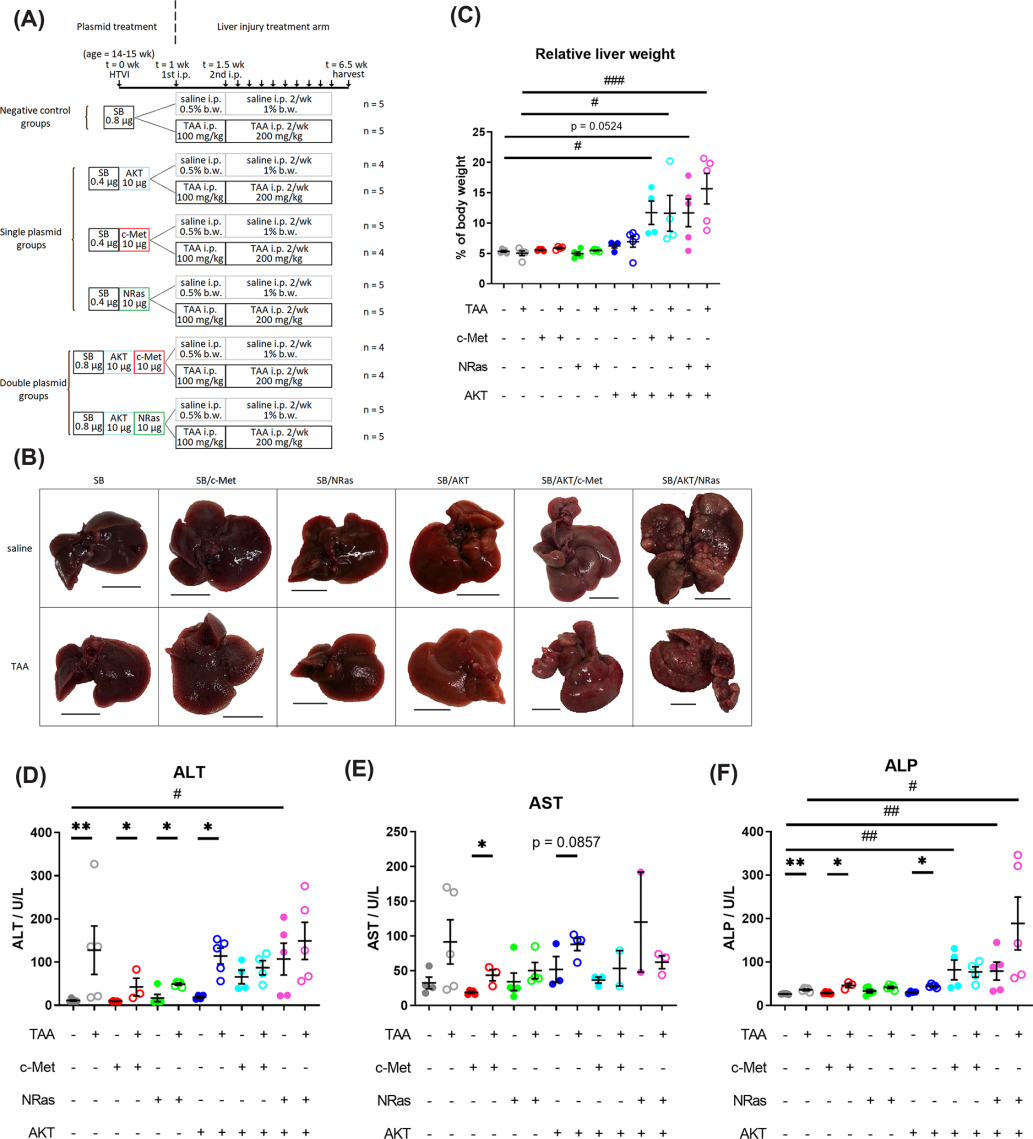
Liver changes following plasmid-HTVI and liver injury (**A**) Schematic of animal experiment cohorts and treatment timeline. Mice received one of six combinations of plasmids by HTVI at 14–15 weeks of age, then began i.p. injections of either saline or TAA dissolved in saline twice per week; the first being half the dose of subsequent doses. (**B**) Representative images of gross appearance of mouse livers on excision; scale bar: 1 cm. (**C**) Relative liver weight at harvest as a percentage of harvest body weight. Data are presented as mean ± SEM. (**D–F**) Liver function tests. Data are presented as mean ± SEM. ALP, alkaline phosphatase; ALT, alanine aminotransferase; AST, aspartate aminotransferase; HTVI, hydrodynamic tail vein injection; i.p., intraperitoneal injection; TAA, thioacetamide; *P<0.05, **P<0.01 (Mann–Whitney test); ^#^*P*<0.05, ^##^*P*<0.01, ^###^*P*<0.001 (Dunn’s post-hoc test).

Plasmids used for the hydrodynamic tail vein injection (HTVI) models of liver cancer were a kind gift from Dr Xin Chen and have been described previously [13,31]. pCMV-SB (‘SB’) plasmid and pT2-CAGGS-NRasV12 (‘NRas’) plasmid were sourced directly from the Chen lab while pT3-EF1a-c-Met (‘c-Met’) and pT3-myr-AKT-HA (‘AKT’) were obtained from Addgene (#31784, #31789, respectively). Endotoxin-free plasmid extracts in nuclease-free water were obtained using the PureLink Expi Endotoxin-Free Maxi Plasmid Purification Kit (Invitrogen, Carlsbad, California, U.S.A.) as per manufacturer’s instructions. Fourteen- to fifteen-week-old male C57BL/6J mice (Australian Bio Resources, Sydney, Australia) were used for liver cancer model experiments. The experimental design and mouse cohorts are shown in Figure 1A. HTVI were performed as previously described [10]. For double oncogene plasmid combinations, the administered dose was 0.8 µg SB plasmid, 10 µg AKT plasmid and 10 µg of either c-Met or NRas plasmid. For single oncogene plasmid combinations, the administered dose was 0.4 µg SB plasmid with 10 µg of AKT, c-Met or NRas plasmid. A 0.8 µg SB plasmid dose was used as a control. Plasmids were resuspended in a volume of 0.9% saline equal to 10% mouse body weight. Mice were included if at least 80% of this volume was injected through the tail vein within 5–7 s. One week after HTVI, mice received an initial intraperitoneal (i.p.) injection of 100 mg/kg body weight thioacetamide (TAA) (Sigma-Aldrich, St Louis, Missouri, U.S.A.) dissolved in saline (0.5% body weight) or an equal volume of saline, followed by either 200 mg/kg body weight TAA in saline (1% body weight) or saline twice per week. The lower initial TAA dose was to allow mice to adjust to the liver injury in order to reduce acute mortality. After receiving 10 or 11 i.p. injections, mice were killed by carbon dioxide asphyxiation.

Mice were monitored in the 30-min period, at four hours and daily for one week post-HTVI for recovery. Afterward, mice were monitored thrice per week (including 30-min post-intraperitoneal injection) and weighed weekly except in cases of ≥5% weight loss (weighing frequency increased to thrice per week). Humane endpoints for euthanasia by carbon dioxide asphyxiation were ≥20% weight loss from baseline before HTVI, or other scoring of clinical examination parameters.

### Liver function test

Serum samples were diluted three-fold in phosphate-buffered saline (PBS) (Lonza, Basel, Switzerland) prior to analysis with a liver function test panel (Sydney South West Pathology Service, Sydney, NSW, Australia).

### Histology

Liver tissue was fixed in 10% neutral-buffered formalin (Sigma-Aldrich) and embedded in paraffin. Five micrometer-thick formalin-fixed paraffin embedded (FFPE) sections were cut for hematoxylin and eosin (H&E) staining and picro-sirius red (PSR) staining.

For Oil Red O and immunofluorescence, liver tissue was embedded in Optimal Cutting Temperature medium (OCT) (Sakura Finetek, Torrance, California, U.S.A.) and slowly frozen in liquid nitrogen vapor phase before storage at −80°C.

### Immunohistochemistry

Antibodies were diluted in 3% bovine serum albumin (BSA) (Sigma-Aldrich) in PBS. Five micrometer-thick FFPE sections were used for immunohistochemical staining for alpha smooth muscle actin (α-SMA). Endogenous peroxidase was blocked with 3% H_2_O_2_/50% methanol in water (10 min). Heat-mediated antigen retrieval was performed using 10 mM sodium citrate (pH 6.0) and bringing to low boil 3–5 times in a microwave. Washes between steps used Tris-buffered saline with 0.05% Tween 20. Non-specific antibody binding was blocked using 10% goat serum (Sigma-Aldrich) in PBS. α-SMA was stained using a monoclonal rabbit anti-mouse primary antibody (ab32575, 1:200 dilution, Abcam, Cambridge, England) incubated 4°C overnight and goat anti-rabbit IgG secondary antibody with horseradish peroxidase (HRP) conjugate (P0448, 1:100 dilution, Dako Agilent, Santa Clara, California, U.S.A.) incubated at room temperature for 45 min, washing with Tris-buffered saline with 0.05% Tween 20 (Sigma-Aldrich; Astral Scientific, Sydney, NSW, Australia). Chromogenic detection was performed using SIGMAFAST 3,3′- Diaminobenzidine tablets (DAB) (Sigma-Aldrich) as per manufacturer’s instructions. Sections were counterstained with Harris’ hematoxylin (Sigma-Aldrich), differentiated with 0.3% v/v acid alcohol and blued with Scott’s tap water substitute. A secondary antibody-only control was performed with the primary antibody replaced with 3% BSA in PBS.

### Immunofluorescence

Seven micrometers thick frozen tissue sections were brought to room temperature over 1 h before fixation/permeabilization in ice-cold 50% acetone/methanol (Sigma-Aldrich, Thermo Fisher Scientific, Waltham, Massachusetts, U.S.A.), drying and washing in PBS (Sigma-Aldrich). Sections were blocked against non-specific antibody binding with 10% donkey serum diluted in PBS for 30 min at room temperature. Antibodies were diluted in 3% w/v BSA in PBS. Sections were stained with primary antibodies against cluster of differentiation (CD)45 (14-0451-82, 1:200 dilution, Invitrogen) and cytokeratin (CK)19 at 4°C overnight (ab52625, 1:200 dilution, Abcam), secondary antibodies at room temperature for 45 min (A21209 and A21206, both 1:200 dilution, Invitrogen) and counterstained with Hoechst 33342 (5 µg/ml in PBS, Invitrogen) for 5 min, with PBS washes in between. Coverslips were mounted using Prolong Diamond mounting medium (Invitrogen) and sealed with nail polish. A secondary antibody-only control was performed with the primary antibody replaced with 3% BSA in PBS.

### Oil Red O staining

Oil Red O staining for lipid was adapted from previously published methods [32]. In brief, 7 µm-thick frozen tissue sections were brought to room temperature over one hour before brief washing in PBS (Sigma-Aldrich). Sections were fixed in 10% neutral buffered formalin (Sigma-Aldrich), stained with freshly prepared Oil Red O stain and counterstained with Mayer’s hematoxylin (Sigma-Aldrich) with rinsing between steps. Coverslips were mounted with Faramount aqueous mounting medium (Dako Agilent, Santa Clara, California, U.S.A.).

### Microscopy

Brightfield images of slides were captured using a Leica DM2000 microscope with DF450C digital camera (Leica Microsystems, Wetzlar, Germany). PSR slides were also imaged using a polarizing filter. Digital images were saved using corresponding software (Leica Application Suite v4.11.0, Leica Microsystems).

For whole-section scanning, H&E-stained sections were using a PreciPoint M8 microscope scanner (PreciPoint, Freising, Germany) with a 20× objective and predictive focus. Image analysis was performed using corresponding software (Viewpoint 1.0.0.9628, PreciPoint). Preneoplastic lesions (clear cell foci) and neoplastic lesions (nodules and tumors) were counted and their diameters measured on section scans. All lesions were pooled for quantitative analysis. Lesion counts were normalized to whole section area measured manually using the whole section scan.

Immunofluorescence-stained sections were imaged using a BX53 microscope with DP73 camera (Olympus, Tokyo, Japan) using preset filters (DAPI (4′,6-diamidino-2- phenylindole); GFP (green fluorescent protein); TRITC (tetramethylrhodamine)). Digital images were recorded using corresponding software (cellSens Dimension 1.6, Olympus).

### Histological grading

The histological scoring systems for steatosis and inflammation were adapted from literature [33]. Steatosis was scored based on proportion of area of field of view affected, regardless of type (macro- or micro-vesicular) (score 0: <5% area, 1: 5–33% area, 2: 34–66% area, 3: 67–100% area). Inflammation was scored based on number of inflammatory foci per field of view (a cluster of five or more inflammatory cells not in a row) (Score 0: <0.5 foci, 1: 0.5–1.0 foci, 2: 1.0–2.0 foci, 3: >2.0 foci). The histological scoring system for fibrosis was based on the Metavir scoring system [34] and other studies [35] and modified to account for morphological differences in fibrosis between groups with or without steatosis. For each animal, five fields of view of H&E or PSR section at 100× magnification were imaged (where possible, excluding preneoplastic or neoplastic lesions), scrambled and scored in blinded fashion on two separate occasions, for a total of ten scores. The median of the scores was considered the histological steatosis or fibrosis grade of each liver, while inflammation score was calculated based on the average count of inflammatory foci.

### Liver tissue triglyceride assay

Hepatic lipids were extracted in chloroform:methanol (2:1 v/v), and the phases were separated with 4 mmol/L MgCl_2_. Triglyceride content was determined by colorimetric assay (Triglycerides GPOPAP; Roche Diagnostics) [36].

### RNA extraction and cDNA synthesis

RNA was extracted from snap-frozen tissue using RNAzol RT (Sigma-Aldrich) according to manufacturer’s instructions and dissolved in nuclease-free water (Sigma-Aldrich). In order to extract RNA from small tumors and adjacent tissue, RNA was instead extracted from fresh-frozen tissue preserved using OCT (Sakura Finetek). Embedded tissue was allowed to soften on ice before surrounding OCT was removed manually with a scalpel. Tissue was washed with clean PBS (Lonza, Basel, Switzerland) to remove residual OCT. Tumor and adjacent tissue was excised with a scalpel. Due to purity and yield concerns, RNeasy Mini Kit (Qiagen, Hilden, Germany) was used for these RNA extractions. Yield and purity were quantified using a Nanodrop 2000 UV spectrophotometer (Thermo Fisher Scientific).

RNA was diluted to 200 ng/µl with nuclease-free water before reverse transcription using a Superscript III kit (Invitrogen) as per manufacturer’s instructions (without oligo-dT primers). Random hexamer primers were sourced from Roche (Basel, Switzerland) and deoxyribonucleotide triphosphates (dNTPs) were sourced from Meridian Biosciences (formerly Bioline). Complementary DNA (cDNA) was diluted 1:5 in nuclease-free water prior to downstream analysis. For no-reverse transcriptase controls, enzyme was replaced with nuclease-free water during the reverse transcription step.

### OpenArray gene expression analysis

Two custom Applied Biosystems OpenArray panels (referred to as Fibrosis and Immune panels) on 56-assay plates were previously designed in our lab (full panel gene lists provided in Supplementary Tables S1 and 2). cDNA was loaded with TaqMan OpenArray Real-Time PCR Master Mix (Applied Biosystems, Waltham, Massachusetts, U.S.A.) on to the plates and analyzed as per manufacturer’s instructions. Data were exported using corresponding software (QuantStudio 12K Flex Real Time PCR System v1.2.2, Applied Biosystems) and analyzed using the delta-delta Ct method [37] using *Hprt* as the housekeeping gene.

### Statistical analysis

To determine differences caused by plasmid treatments (considering saline and TAA groups separately), statistical significance was determined using a Kruskal–Wallis test and Dunn’s post hoc test, comparing to the respective SB + saline or SB + TAA control. To determine differences caused by TAA, statistical significance was determined using two-tailed Mann–Whitney tests comparing plasmid + TAA groups against their respective saline counterparts. For nested data, comparisons between plasmid + TAA groups and saline counterparts were made using a nested *t*-test. A *P*-value < 0.05 was considered significant. Statistical analysis was performed using GraphPad Prism 7 and 8 (GraphPad Software, San Diego, California, U.S.A.).

### RNA sequencing

For RNA sequencing (RNA-seq), all RNA were extracted from snap-frozen bulk liver tissue with RNAzol according to manufacturer’s instructions and dissolved in nuclease-free water. Quality control and the RNA-seq reaction were performed by the Monash Health Translation Precinct Medical Genomics Facility. RNA-seq was performed as described by Grubman and colleagues [38]. Briefly, samples were given a unique index (together with unique molecular identifier (UMI)) during individual pA priming and first strand synthesis which also adds a template switch sequence to the 5′-end. Samples were then pooled and amplified using an oligo which binds the index primer and template switch sequence. Final library construction is completed by Nextera tagmentation and addition of Illumina P7 and P5 sequences. Sequencing was performed on an Illumina NextSeq550 run (Illumina, San Diego, California, U.S.A.) with a custom R1 primer to generate an 18nt R1 which contains the 8nt index and 10nt UMI. A standard R2 primer was then used to sequence the cDNA in sense orientation. The dataset can be found on the NCBI Gene Expression Omnibus database (accession ID 174074).

### RNA sequencing data analysis

Raw read processing was performed by the Monash Bioinformatics Platform. Samples were demultiplexed using Sabre (doi:10.21105/jose.00053). Demultiplexed raw reads were aligned to a reference mouse genome (GRCm38) modified with additional human transgenes (NRAS, NM_002524.5; MET, NM_000245.4). Read alignment, post-processing, quantification and QC were performed using the RNAsik pipeline [39] (doi:10.5281/zenodo.1403976). As part of RNAsik, reads were aligned to the reference genome using STAR aligner [40], reads were filtered and deduplicated by UMI sequences using Picard (https://broadinstitute.github.io/picard/) and Samtools [41] and read quantification was performed using featureCounts [42]. Library QC data were aggregated using MultiQC [43] Raw gene counts were uploaded to Degust [44] (doi:10.5281/zenodo.3258932) with a threshold of ≥0.5 counts per million in at least two samples for differential expression analysis and multidimensional scaling. Differential expression analysis was performed in Degust using the voom/limma method [44]. Differentially expressed genes were determined both on multiple comparisons (considering all groups) and on pairwise comparisons between cancer plasmids without TAA versus negative control (SB + saline), TAA alone versus negative control and the cancer plasmids-TAA combinations versus cancer plasmids without TAA or TAA alone. Except where otherwise stated, genes with a false discovery rate (FDR) < 0.05, and >2-fold change (abs log2FC > 1) were considered differentially expressed. For enrichment analysis of pairwise comparisons, gene lists were separated into up-regulated and down-regulated relative to control (>2-fold change) and the FDR < 0.05 filter was applied for both multiple comparison and pairwise comparison. Differentially expressed gene lists were input into gProfiler and Panther [45,46] for pathway analysis. An adjusted *P*-value (gProfiler) or FDR < 0.05 (Panther) was considered significant. For Gene Set Enrichment Analysis (GSEA), gene lists were ordered using a ranking metric (sign(log2FC) × -log10(FDR)) and input into the GSEA-preranked module on GenePattern [47]. Pathways enriched at FDR < 0.25 level were considered significant [48]. The Hallmark and Canonical Pathways gene sets used for GSEA were accessed through the Molecular Signatures Database [49–54].

## Results

### Tumors develop on an injury background but are phenotypically different

In order to induce liver cancer, mice were given hydrodynamic tail vein injections of plasmids to express constitutively active forms of AKT1 and NRas (SB/AKT/NRas), or AKT1 and c-Met (SB/AKT/c-Met) (Figure 1A). To study the effect of concomitant liver injury on the cancer model phenotype, TAA was administered for six weeks with saline as control. We have not performed a dose response study with the plasmids and/or TAA. The plasmid dosage was based on the original AKT/NRas and AKT/c-Met papers [13,31], while TAA dosage was based on our previous laboratory experience of using TAA up to 8 weeks to induce liver injury without neoplastic changes.

### Liver injury model

**Figure 2:**
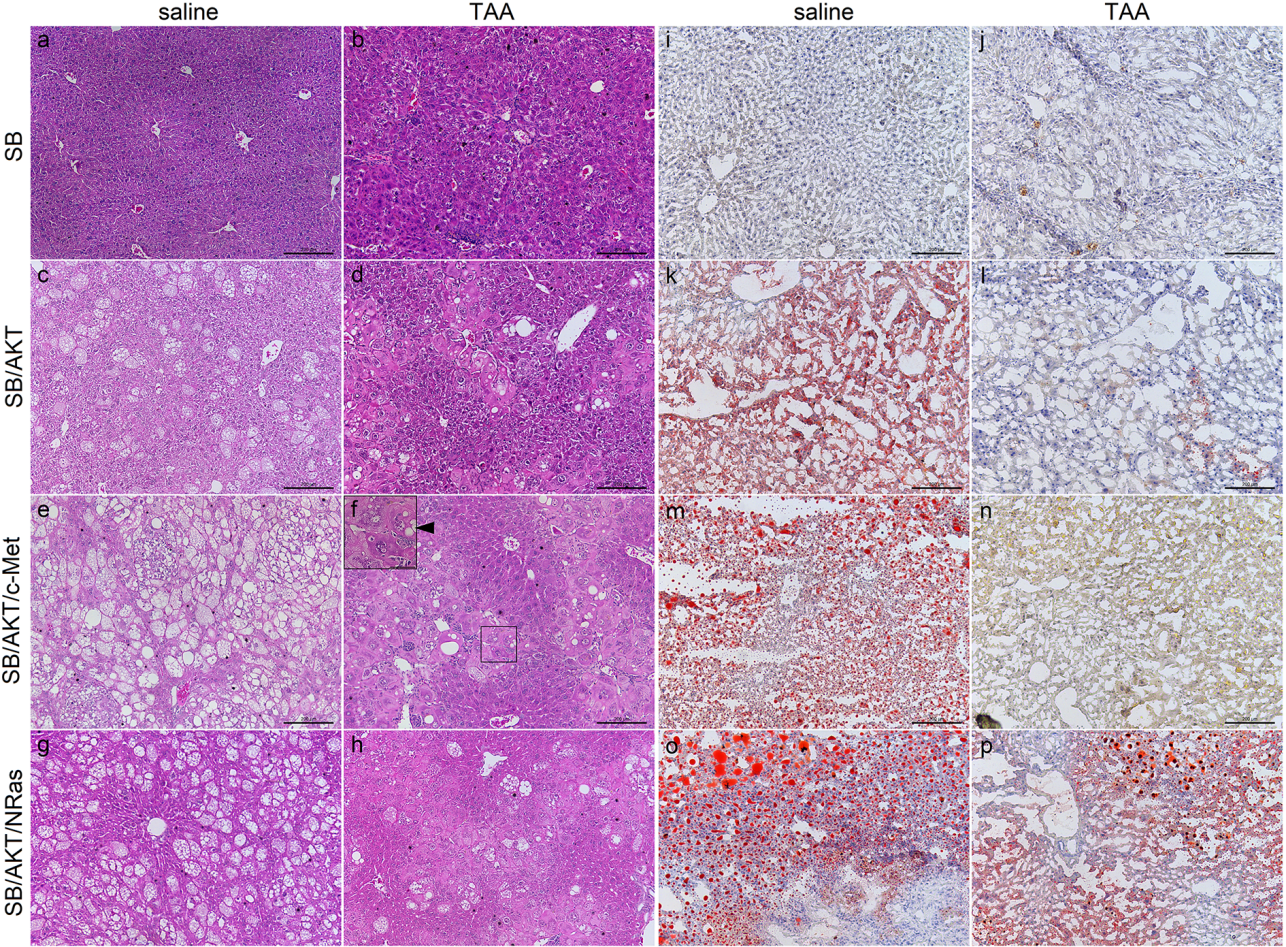
Plasmid-HTVI and liver injury are associated with changes in hepatic lipid content and distribution (**A–H**) Hematoxylin and eosin staining of mouse livers, representative non-tumor fields of view; scale bar: 200 µm. Representative high-power image of a large cell is shown in (**F**) (inset); scale bar = 50 µm. (**I–P**) Oil Red O staining of mouse livers, representative non-tumor fields of view; scale bar: 200 µm. Batch variation of Oil Red O stain preparation caused different colouration in (**N**) but no change in distribution. Liver sections from SB/c-Met and SB/NRas plasmid groups appeared similar to SB-alone counterparts with H&E staining (Supplementary Figure S1) and Oil Red O staining (Supplementary Figure S9); TAA, thioacetamide, ►, vacuolization (F).

TAA alone (SB + TAA) produced morphological and histological changes consistent with chronic liver injury as previously described [55]. On gross examination, a rough surface texture consistent with fibrosis was observed, but no tumor nodules (Figure 1B). Serum biochemical markers of liver injury alanine aminotransferase (ALT) and alkaline phosphatase (ALP) were significantly increased by TAA (Figure 1D–F). In hematoxylin and eosin (H&E) stained liver sections, disrupted lobular architecture with mild centrilobular necrosis, pericentral mononuclear infiltrates and eosinophilic central–central bands were observed (Figure 2B) compared with SB + saline which had normal liver histology (Figure 2A).

### Single plasmid models with and without TAA administration

As described in previous studies [13,31], single oncogene plasmids were unable to induce tumor development. Notably, this was true even in the presence of liver injury. None of the single oncogene plasmid groups showed gross abnormalities, although SB/AKT had a pale external surface relative to their SB-alone counterparts (Figure 1B). All single oncogene plasmid groups showed elevated ALT due to TAA, although aspartate aminotransferase (AST) and ALP were elevated in some groups (Figure 1D–F). Histologically, SB/c-Met and SB/NRas with and without injury appeared identical to their SB-alone counterparts (Supplementary Figure S1). On the other hand, SB/AKT + saline showed steatosis as previously reported [56] (Figure 2C). Interestingly, in SB/AKT combined with TAA, steatosis was almost completely absent and replaced with pleiomorphic hypertrophic cells (henceforth referred to as large cells) which resembled the amphophilic focus described by Thoolen and colleagues [57] (Figure 2D,F inset). Large cells were located pericentrally, had a normal nuclear-to-cytoplasmic ratio, but sometimes had indistinct nuclei and poorly-demarcated basophilic staining. Other features included vacuolization and less eosinophilic staining compared with normal hepatocytes. Consistent with gross pathology, no neoplastic lesions were observed in any single oncogene plasmid groups.

### Dual plasmid models with and without TAA administration

Two oncogenes were necessary for tumor development. Unlike the original studies [13,31], mice did not exhibit clinical signs of tumor-induced ill-health warranting euthanasia up to time of sacrifice at seven weeks post-HTVI. One mouse in the SB/AKT/NRas + TAA group had >10% weight loss from baseline. At sacrifice, livers from SB/AKT/c-Met + saline mice were enlarged and had pale external surface relative to the SB plasmid without TAA control (SB + saline), but had only few macroscopic nodules of similar color to background liver (Figure 1B). In SB/AKT/NRas + saline, three out of five mice had enlarged livers with pale external surface relative to SB + saline, with multifocal clusters of macroscopic nodules. One mouse had few nodules 1–2 mm in diameter and one mouse had no visible nodules (Figure 1B).

The effect of concurrent liver injury on cancer development varied between models. Similar to its saline counterpart, livers from the SB/AKT/c-Met + TAA group had few macroscopic nodules and were enlarged with pale external surface relative to SB + TAA (Figure 1B). Surprisingly, livers from the SB/AKT/NRas + TAA group had a diffuse miliary distribution of smaller nodules (majority <2 mm diameter) (Figure 1B). Liver weights at harvest (relative to body weight) were significantly increased in all double oncogene groups compared with the respective SB-alone groups (Figure 1C). Some dual plasmid models without TAA showed some abnormal liver function tests, although there was no further significant increase due to TAA (Figure 1D–F).

**Figure 3:**
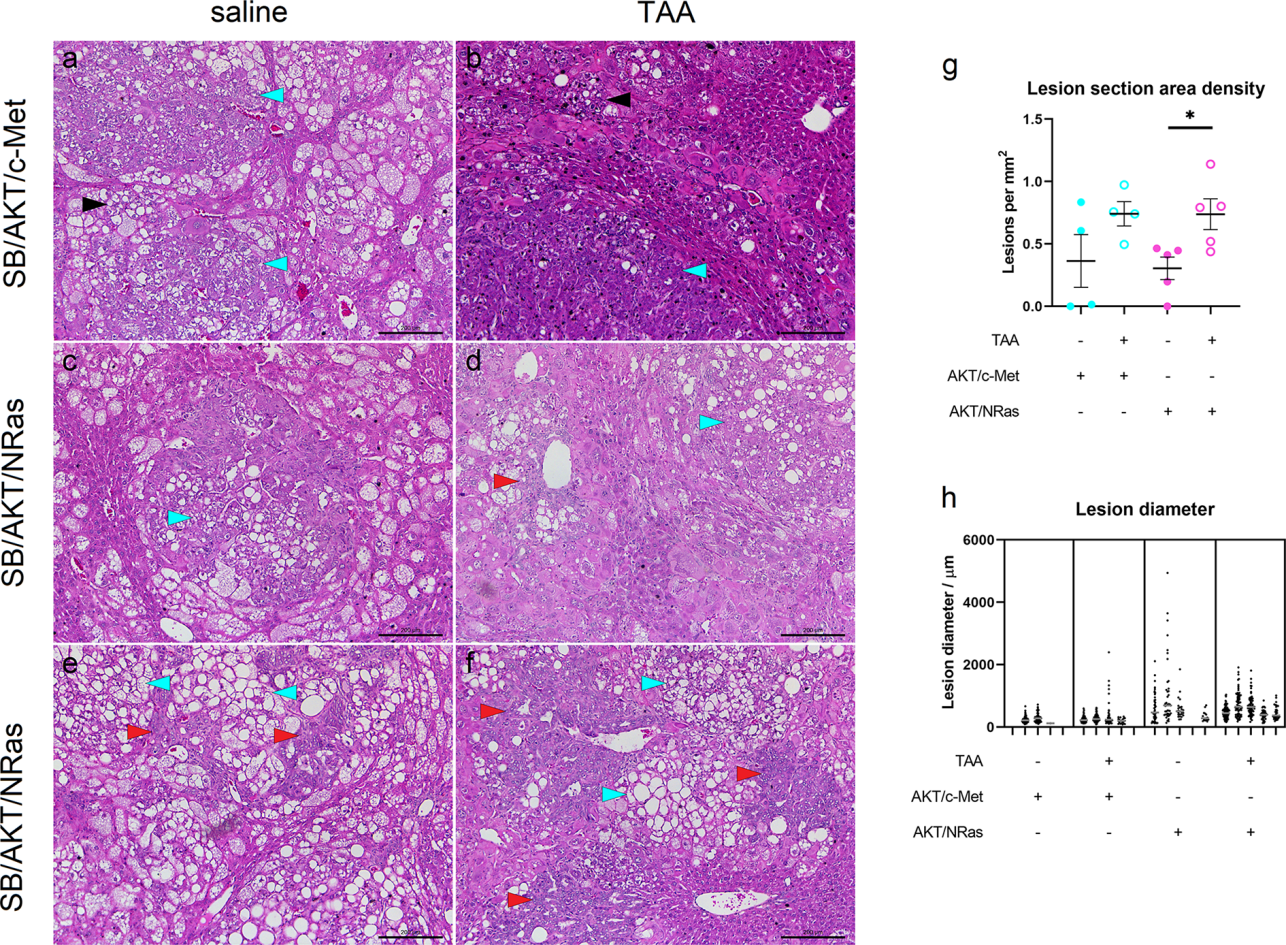
Neoplastic lesions form in SB/AKT/c-Met and SB/AKT/NRas groups with and without liver injury (**A–F**) Hematoxylin and eosin staining of mouse livers, representative tumors or pre-neoplastic lesions; scale bar = 200 µm. (**G**) Quantitative histological measurements of lesion number in cancer models. Data are presented as mean ± SEM (**H**) Quantitative histological measurements of lesion size in cancer models. Each individual dot plot represents one mouse; each dot is a single lesion diameter measurement. There were no significant differences between saline and TAA arms for each plasmid group (nested *t*-test). Grey line shows median of each distribution; TAA, thioacetamide; *P<0.05, Mann–Whitney; 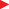, cholangiocellular neoplastic lesion; 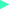, hepatocellular neoplastic lesion; ►, clear cell focus.

Examination of H&E-stained liver sections from SB/AKT/c-Met + saline animals showed preneoplastic foci of clear cells consistent with previous descriptions [31,56] in three out of four mice. In at least one mouse, there were neoplastic hepatocellular nodules with a mixture of lipid-rich and basophilic lipid-poor cells consistent with previous descriptions of this model [31], although tumors with invasive borders were not observed (Figure 3A and Supplementary Figure S2). Similar preneoplastic and neoplastic lesions were observed in all mice receiving SB/AKT/c-Met + TAA, with one additionally having a high-grade trabecular HCC (Figure 3B and Supplementary Figure S2).

In animals receiving SB/AKT/NRas with and without TAA, nodules and tumors of mixed cellularity were observed, including clear cell hepatocellular, cholangiocellular and mixed hepatocellular-cholangiocellular lesions (Figure 3C–F and Supplementary Figure S2). Immunofluorescent staining of cytokeratin (CK)19, which is expressed in liver progenitors and biliary epithelium [58] and a subtype of HCC [59], is also consistent with cholangiocellular tumors in SB/AKT/NRas groups (Supplementary Figure S3). Non-specific immunostaining or autofluorescence was not observed in secondary antibody-only controls (Supplementary Figure S4).

To corroborate the observed tumor gross morphology, whole-section scans were analyzed to quantify the size and number of preneoplastic and neoplastic lesions. For all samples, the majority of lesions were microscopic (<500 µm diameter), although there was heterogeneity in size and number within each group. There was a 2.4-fold increase in density of lesions in SB/AKT/NRas + TAA compared with SB/AKT/NRas + saline (Figure 3G) but no significant difference in lesion diameter (Figure 3H). As a further investigation, lung tissue was examined for metastasis. No obvious lung metastases could be found on gross examination or in any histological sections (data not shown).

### Liver fibrosis and inflammation in dual plasmid models with and without TAA administration

**Figure 4:**
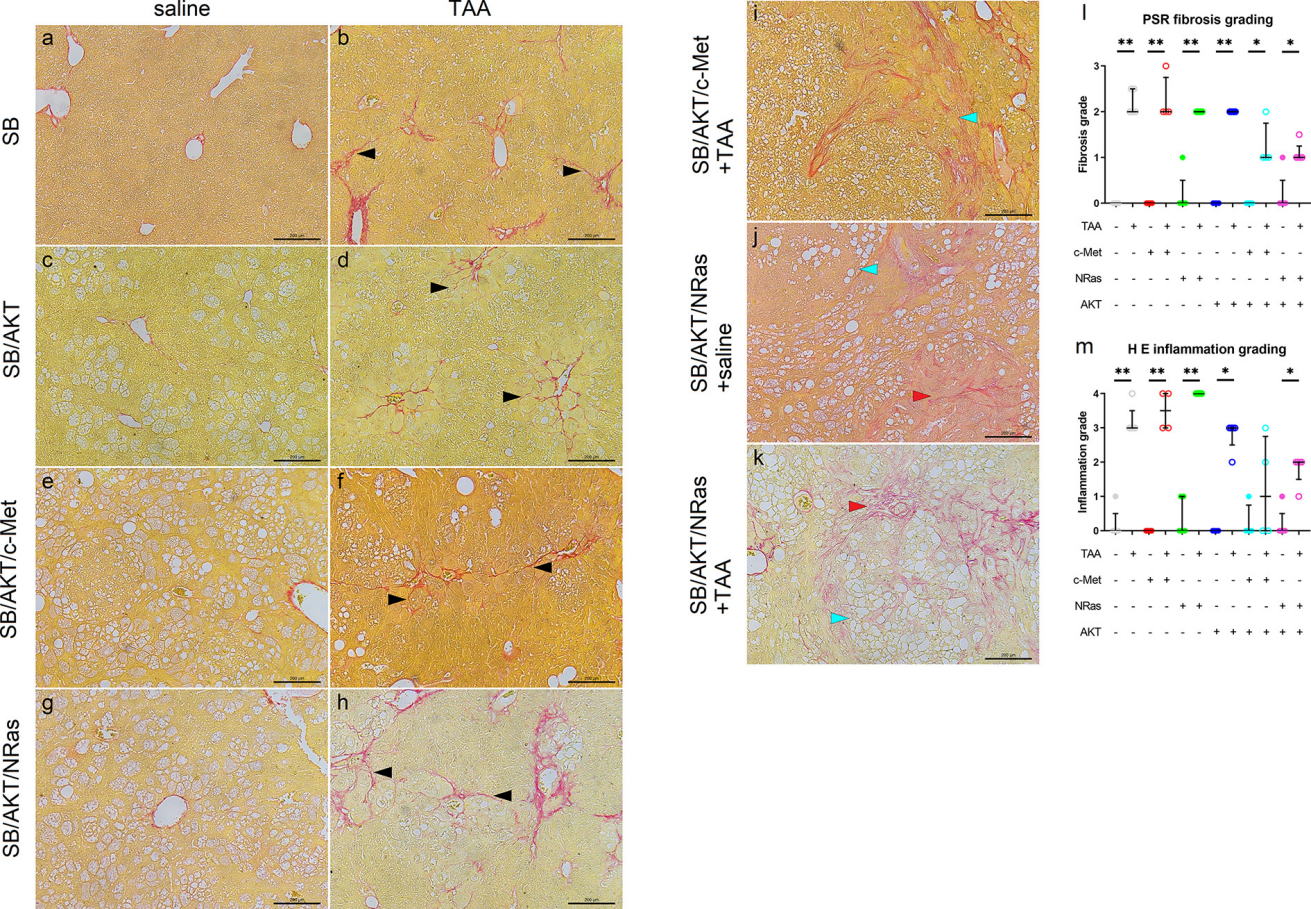
Fibrosis and inflammation occur in the presence of liver injury and cholangiocellular neoplasms (**A–H**) Picro-sirius red staining of mouse livers, representative non-tumor fields of view; scale bar = 200 µm. Liver sections from SB/c-Met and SB/NRas plasmid groups appeared similar to SB-alone counterparts with Picro-sirius red staining (Supplementary Figure S5). (**I–K**) Picro-sirius red staining of mouse livers, representative tumors. (l) Semi-quantitative histological grading of fibrosis. Data are presented as median ± interquartile range. (**M**) Semi-quantitative histological grading of inflammation. Data are presented as median ± interquartile range. H&E, hematoxylin and eosin; PSR, Picro-sirius red; TAA, thioacetamide; *P<0.05, **P<0.01 (Mann–Whitney test); 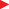, cholangiocellular neoplastic lesion; 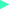, hepatocellular neoplastic lesion; ►, fibrous septa (B) and pericellular fibrosis (D, F, H).

As liver fibrosis is a common precursor condition to liver cancer, picro-sirius red (PSR) staining was performed to determine the presence and localization of collagen. As expected, fibrosis was primarily associated with TAA-induced liver injury, although the pattern was altered in some groups. With TAA alone, fibrous septa with some central-central bridging fibrosis were observed as previously described [55] (Figure 4B). Consistent with the histopathology, SB/c-Met + TAA and SB/NRas + TAA appeared identical to SB + TAA (Supplementary Figure S5). On the other hand, fibrosis in SB/AKT + TAA (Figure 4D) was primarily pericellular. In non-cancer groups without liver injury, abnormal collagen deposition was not observed (Figure 4A,C and Supplementary Figure S5).

In the absence of liver injury, the double oncogene plasmid groups did not show fibrosis in non-tumor tissue or in hepatocellular tumors (Figure 4E,G). However, fibrosis was observed within cholangiocellular tumors in SB/AKT/NRas-injected animals (Figure 4J), consistent with desmoplastic stroma [16]. With liver injury, fibrosis was observed surrounding the high-grade HCC in the SB/AKT/c-Met + TAA group and within cholangiocellular tumors in SB/AKT/NRas + TAA (Figure 4I,K). Outside of tumors, pericellular fibrosis was observed similar to SB/AKT + TAA (Figure 4F,H). Histological grading of fibrosis confirmed that TAA caused significant fibrosis in all groups compared with saline-injected animals (Figure 4L). Ductular reaction was not observed in any groups based on CK19 immunofluorescent staining (Supplementary Figure S6). Polarized light microscopy confirmed the specificity of staining for collagen (Supplementary Figure S7).

**Figure 5:**
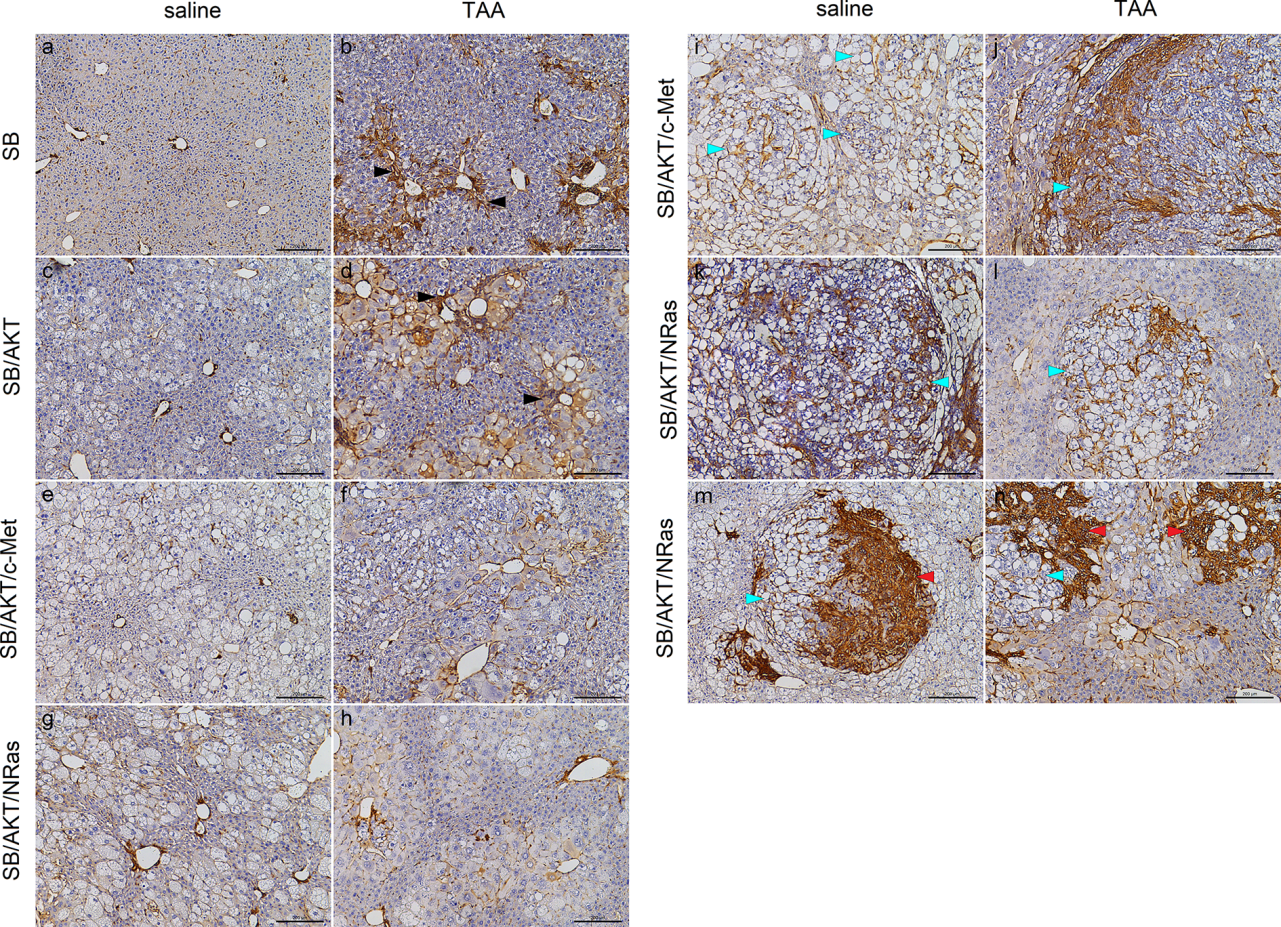
Alpha smooth muscle actin indicates presence of activated hepatic stellate cells (**A–H**) α-SMA immunohistochemical staining of mouse livers, representative non-tumor fields of view; scale bar = 200 µm. Liver sections from SB/c-Met and SB/NRas plasmid groups appeared similar to SB-alone counterparts with α-SMA immunohistochemical staining (Supplementary Figure S8) (**I–N**) α-SMA immunohistochemical staining of mouse livers, representative tumors; scale bar = 200 µm. TAA, thioacetamide; 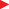, cholangiocellular neoplastic lesion; 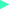, hepatocellular neoplastic lesion; ►, α-SMA-staining septa (B) and pericentral staining (D).

Alpha smooth muscle actin (α-SMA) is a marker for myofibroblasts. In the context of liver fibrosis, α-SMA is also a marker for activated hepatic stellate cells, which are responsible for deposition of extracellular matrix in liver fibrosis. Similar to collagen staining, strong α-SMA staining was present around central veins, forming septa and occasional central–central bridging in mice receiving TAA (Figure 5B and Supplementary Figure S8), with the exception of SB/AKT + TAA with primarily pericentral staining. The cytoplasm of adjacent hepatocytes appeared weakly stained which is consistent with staining of adjacent stellate cells in the perisinusoidal space rather than hepatocyte staining (Figure 5B,D,F,H and Supplementary Figure S8). On the other hand, in mice receiving saline, only normal α-SMA staining was observed around periportal hepatic arteries and sparse isolated cells within sinusoids (Figure 5A,C,E,G).

In both double oncogene plasmid groups, intratumoral α-SMA staining was observed with and without injury. In SB/AKT/c-Met + saline, staining was observed within clear cell foci and nodules, sometimes forming luminal structures (Figure 5I). In SB/AKT/NRas + saline, in addition to staining similar to SB/AKT/c-Met + saline, there was also strong staining within cholangiocellular tumors (Figure 5K,M). Tumor staining in SB/AKT/c-Met + TAA and SB/AKT/NRas + TAA (Figure 5J,L,N) was similar to that of saline counterparts, although strong staining was observed within and around the high-grade HCC.

Inflammatory cell infiltration is another feature of liver injury. Consistent with this, pericentral mononuclear infiltrates were observed in all TAA groups. This was corroborated with the presence of white blood cells as detected by cluster of differentiation (CD)45 immunofluorescent staining (Supplementary Figure S6). TAA caused significantly increased histological inflammation grade except in the SB/AKT/c-Met groups (Figure 4M).

### Tissue lipid in dual plasmid models with and without TAA administration

**Figure 6:**
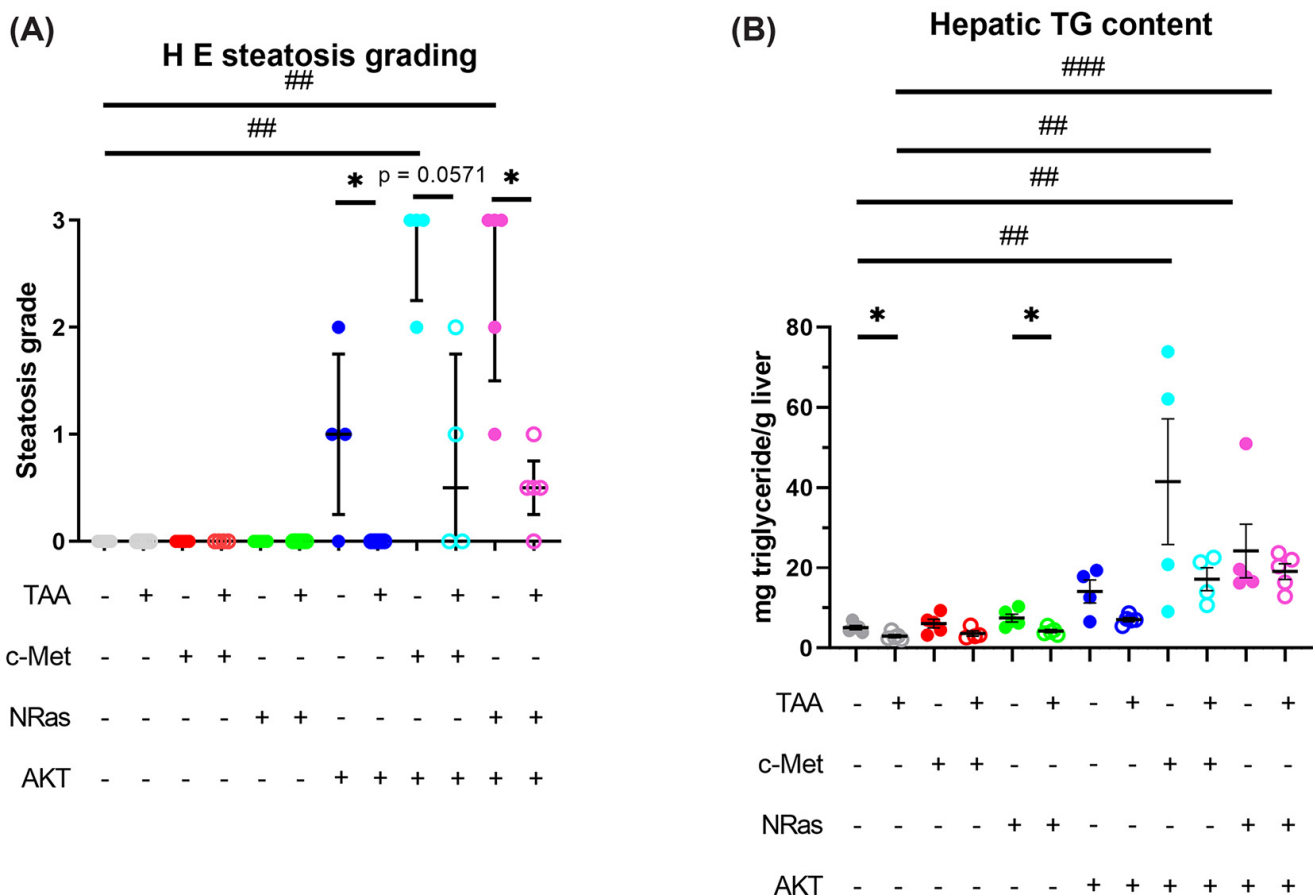
Tissue triglyceride assay reveals changes in hepatic lipid distribution underlying histological changes (**A**) Semi-quantitative histological grading of steatosis. Data are presented as median ± interquartile range. (**B**) Triglyceride assay of liver tissue. Data are presented as mean ± SEM. H&E, hematoxylin and eosin; TAA, thioacetamide; TG, triglyceride; *P<0.05, **P<0.01 (Mann–Whitney test); ^##^*P*<0.01, ^###^*P*<0.001 (Dunn’s post-hoc test).

Hepatic lipid accumulation is a feature of MASLD, which is an increasingly important risk factor for HCC [5]. As previous studies of these plasmid-HTVI cancer models reported steatosis, characterization of hepatic lipid was undertaken. Examination of non-tumor regions of H&E-stained slides found extensive steatosis with hypertrophy in both SB/AKT/c-Met + saline and SB/AKT/NRas + saline (Figure 2E,G) as previously reported [13,31]; histological steatosis score was significantly increased (Figure 6A). Lipid content was confirmed by use of Oil Red O staining (Figure 2I**–**P and Supplementary Figure S9).

With TAA administration, steatosis in double oncogene plasmid groups was reduced and replaced with large cells as seen in SB/AKT + TAA (Figure 2D,F,H). Oil Red O staining found that lipid staining was reduced by TAA, although not completely eliminated (Figure 2L,N,P).

To corroborate the changes in lipid accumulation observed histologically, frozen liver tissue was assayed for triglyceride content. Increased liver tissue triglyceride was observed in SB/AKT/c-Met and SB/AKT/NRas groups compared with SB-alone groups, both with or without TAA (Figure 6B). However, TAA-induced reduction in liver triglyceride was only observed in the SB-alone group and SB/NRas (Figure 6B). This corroborated our finding from the transcriptomic analysis of TAA indicating reduced adipogenesis and fatty acid metabolism pathway activity (see Supplementary Figures S14 and 15).

### Transcriptome expression in plasmid-HTVI and liver injury are associated with changes in pro-fibrotic and pro-inflammatory genes

To investigate fibrosis and inflammation at the gene expression level, real-time PCR was performed with two 28-gene panels (referred to as the fibrosis gene panel (Supplementary Table S1) and the immune gene panel (Supplementary Table S2). Tissue from non-cancer groups were compared with non-tumor tissue from the cancer groups, while a separate comparison of tumor tissue and paired non-tumor tissue was also performed.

Changes in profibrotic and proinflammatory genes were observed due to TAA alone. From the fibrosis gene panel, TAA induced significant changes in nine genes in non-cancer groups (Supplementary Figure S10). While most genes were only up-regulated in one or two groups, collagen I (*Col1a1*), which is the most abundant collagen in liver fibrosis, was a notable exception, being up-regulated at least 5-fold by TAA in all non-cancer groups. From the immune gene panel, 11 genes were significantly altered (Supplementary Figure S11). Interestingly, most genes were up-regulated in multiple groups; genes for chemokines (*Ccl2, Ccl5*), cell adhesion (*Icam1, Lgals3*) and a growth factor (*Ngf*) were up-regulated in at least three non-cancer groups.

Double oncogene plasmid groups without injury showed minimal differential gene expression, suggesting that it lacked an injury microenvironment like that induced by TAA. Only two genes (*Alb, Ccl5*) were significantly altered by either SB/AKT/NRas or SB/AKT/c-Met (Supplementary Figures S10 and 11).

In contrast, the cancer plasmid-TAA combinations had more differentially expressed genes. From the fibrosis gene panel, 10 genes were up-regulated in the SB/AKT/NRas + TAA group compared with either TAA alone or SB/AKT/NRas alone. Notable genes included matrix metalloproteases (*Mmp9, Mmp14*), collagen I (*Col1a1*) and hypoxia inducible factor 1a (*Hif1a*) (Supplementary Figure S10). Only three genes (*Ccr3*, *Lgals3*, *Mif*) from the immune gene panel were altered by either SB/AKT/c-Met + TAA or SB/AKT/NRas + TAA. Notably, *Lgals3* which was up-regulated by TAA in non-cancer groups was also up-regulated in SB/AKT/c-Met + TAA (3.8-fold) and SB/AKT/NRas + TAA (4.1-fold). These data suggest that, at least in the non-tumor tissue, the double oncogene plasmids had little effect on profibrotic and proinflammatory signalling, while the addition of TAA stimulated these pathways in the presence of plasmid.

**Figure 7:**
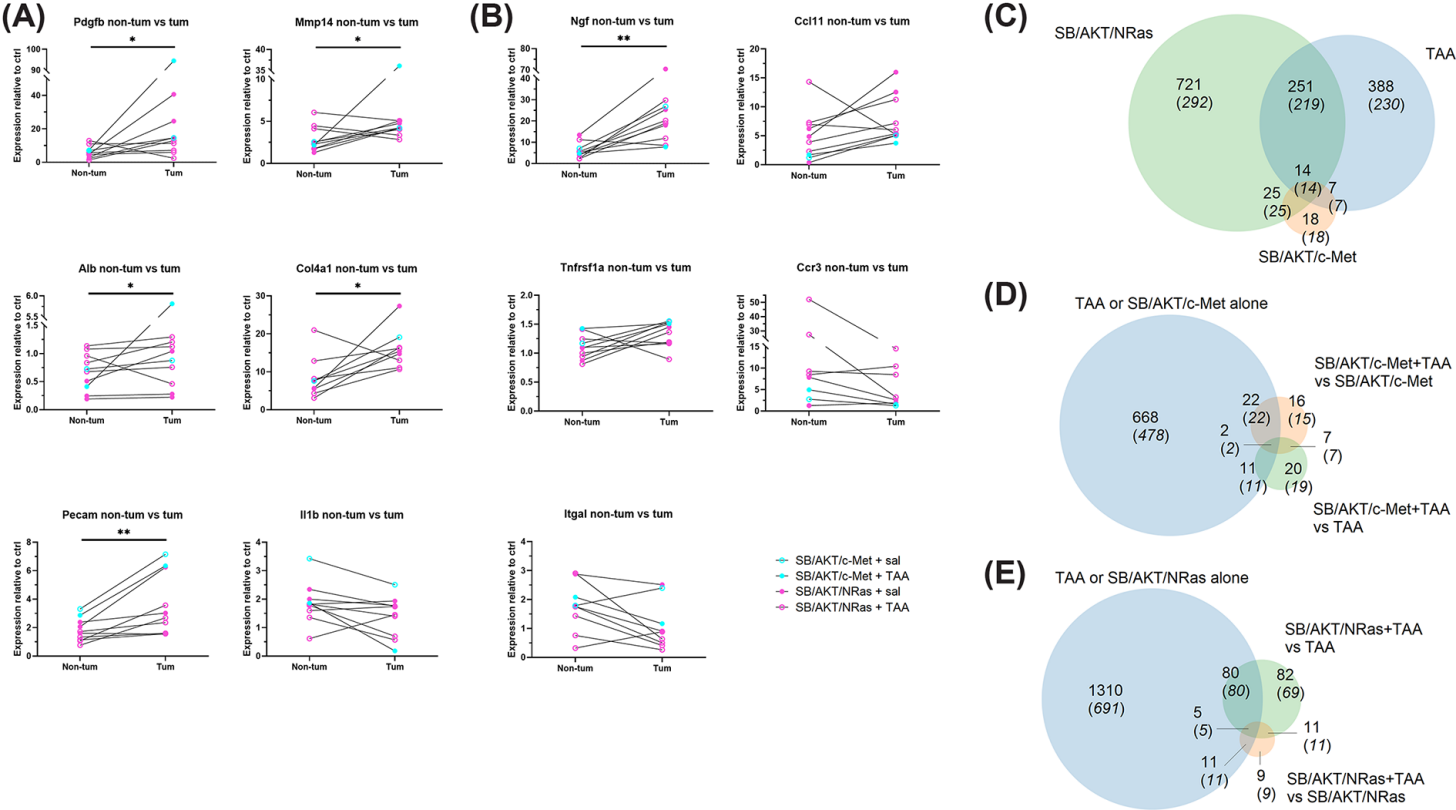
Gene expression changes associated with plasmid-HTVI (**A**) Altered genes between paired tumor and non-tumor tissue, OpenArray fibrosis gene panel. Gene expression shown as fold-change relative to gene expression in SB + saline control group. (**B**) Altered genes between paired tumor and non-tumor tissue, OpenArray immune gene panel. Gene expression shown as fold-change relative to gene expression in SB + saline control group. Panels (A and B) share the same legend for treatment groups. (**C**) Venn diagram showing number of genes differentially expressed by SB + TAA, SB/AKT/c-Met + saline and SB/AKT/NRas + saline compared with SB + saline. (**D**) Venn diagram showing number of genes differentially expressed by SB/AKT/c-Met + TAA compared with SB + TAA or SB/AKT/c-Met + saline. (**E**) Venn diagram showing number of genes differentially expressed by SB/AKT/NRas + TAA compared with SB + TAA or SB/AKT/NRas + saline. *P<0.05, **P<0.01 (Wilcoxon signed rank test).

On comparison of paired tumor versus non-tumor tissue from the same livers, the fibrosis gene panel showed five genes significantly up-regulated in tumors (*Pdgfb*, *Mmp14*, *Alb*, *Col4a1* and *Pecam*). *Il1b* was decreased in 8 out of 10 paired comparisons, but this did not reach statistical significance (*P*=0.0840) (Figure 7A). From the immune gene panel, one gene was significantly up-regulated in tumors (*Ngf*). *Ccl11* and *Tnfrsf1a* were increased in 8 out of 10 paired comparisons, but these did not reach statistical significance (*P*≤0.0840). *Ccr3* and *Itgal* were reduced in 6 out of 8 and 7 out of 9 paired comparisons, respectively, but these did not reach statistical significance (*P*≤0.0781) (Figure 7B).

### Distinct transcriptomic profile in cancer plasmids-TAA combinations compared with cancer plasmids without TAA or TAA alone

For a more comprehensive characterization of gene expression in these models, RNA sequencing (RNA-seq) was performed. Twenty-four samples from bulk liver tissue of SB, SB/AKT/c-Met and SB/AKT/NRas (with or without TAA) were analyzed. Of 19381 genes detected, 1363 were differentially expressed on multiple comparisons (false discovery rate [FDR] < 0.05). Of these, 1076 had >2-fold change on any pairwise comparison. Expression of the human *NRAS* and *MET* transgenes was also confirmed. Hierarchical clustering (Supplementary Figure S12) and multi-dimensional scaling (Supplementary Figure S13) found that SB/AKT/c-Met + TAA and SB/AKT/NRas + TAA formed a distinct cluster; the cancer plasmids-TAA combinations were more similar to each other than to their respective cancer plasmids without TAA or TAA alone groups.

Among cancer plasmids without TAA or TAA alone treatments, pairwise comparison with the negative control found that SB/AKT/NRas + saline had the most differentially expressed genes (550 after multiple comparisons correction), followed by SB + TAA (470) and SB/AKT/c-Met + saline (64) (Figure 7C). Of note, 14 differentially expressed genes were shared between all three (Supplementary Table S3), while 25 were shared between SB/AKT/NRas + saline and SB/AKT/c-Met + saline (Supplementary Table S4), which may relate to a common pathogenic process.

In contrast, pairwise comparisons between cancer plasmids-TAA combination groups and respective cancer plasmids without TAA or TAA alone groups yielded fewer differentially expressed genes. Approximately 54% of differentially expressed genes found in the SB/AKT/c-Met + TAA versus SB/AKT/c-Met + saline or SB/AKT/c-Met + TAA versus SB + TAA comparisons were not altered by either cancer plasmids without TAA or TAA alone compared with negative control (Figure 7D; Supplementary Tables S5 and 6), while 48% of genes found for similar comparisons with SB/AKT/NRas were unique to the cancer plasmids-TAA combination (Figure 7E; Supplementary Tables S7 and 8).

### Pathway analysis identifies differences in metabolic pathways with dual plasmid models

In order to make biological interpretations of the large lists of differentially expressed genes, pathway analysis was performed. gProfiler and Panther were used to analyze selected lists of differentially expressed genes, while Gene Set Enrichment Analysis (GSEA) considered the changes of all genes. TAA alone up-regulated pathways primarily related to cell cycle, mitosis and replication, with some xenobiotic metabolism-related genes. Down-regulated pathways were primarily metabolic, including lipid, amino acid, xenobiotic and steroid metabolism (Supplementary Table S9). From the Molecular Signatures Database Hallmark gene sets, pathways related to inflammation (allograft rejection, inflammatory response, interferon gamma response, TNF alpha signaling) and cell proliferation (DNA repair, G2M checkpoint, up in KRAS signaling, mitotic spindle, MTORC1 signaling) were up-regulated, while pathways related to physiological liver functions (bile acid metabolism, coagulation, Wnt/β-catenin signaling, xenobiotic metabolism) were down-regulated (Supplementary Table S10). These findings are consistent with chronic injury causing cell death, loss of physiological function and compensatory cell division.

There were differences between the cancer plasmids without TAA groups in terms of perturbed pathways. SB/AKT/NRas alone up-regulated cell cycle pathways and down-regulated metabolic pathways similar to TAA alone, but also up-regulated signal transduction and down-regulated nucleotide metabolic pathways (Supplementary Table S11). On the other hand, SB/AKT/c-Met alone had only one up-regulated pathway (response to stilbenoid [GO:0035634]), which may be due to the relatively low number of differentially expressed genes (Supplementary Table S11). By GSEA, both SB/AKT/NRas and SB/AKT/c-Met without TAA shared with TAA alone perturbed pathways relating to inflammation, cell proliferation and physiological liver function, although only SB/AKT/c-Met had up-regulated lipid metabolism pathways (adipogenesis, fatty acid metabolism and peroxisome) (Supplementary Table S10, Supplementary Figures 14 and 15). Notably, some inflammation-related pathways were up-regulated exclusively by both cancer plasmid models without TAA but not TAA alone, including IL2/STAT5 signaling, IL6/JAK/STAT3 signaling, and interferon alpha response (Supplementary Table S12).

To determine pathways that may be involved in the interaction between cancer and injury, comparisons were made between cancer plasmid-TAA combinations and either cancer plasmids without TAA or TAA alone. gProfiler and Panther were less sensitive for this purpose, likely due to the smaller number of differentially expressed genes and the fact that relevant pathways were already perturbed by cancer plasmids without TAA or TAA alone. On the other hand, GSEA identified more pathways altered in the cancer plasmid-TAA combinations, although most were already perturbed to some extent by cancer plasmids without TAA or TAA alone. Fifteen pathways were altered by SB/AKT/c-Met + TAA, either significantly different from SB/AKT/c-Met alone or TAA alone, or uniquely altered in the combination (Table 1). Some showed cooperative up-regulation by both SB/AKT/c-Met and TAA (late estrogen response, IL6/JAK/STAT signaling, p53 pathway), but most (including adipogenesis and fatty acid metabolism) showed opposing trends (Supplementary Figures S14 and 15). Twenty-two pathways were altered by SB/AKT/NRas + TAA by the same criteria (Table 2). MTORC1 signaling, p53 pathway, glycolysis, hypoxia and late estrogen response showed cooperative up-regulation by cancer and injury. Surprisingly, some pathways (including fatty acid metabolism) were down-regulated by both TAA alone and SB/AKT/NRas alone but up-regulated in the combination (Supplementary Figures S14 and 15). These trends suggest that while cancer and injury may act on similar pathways, the combination may have a complex interaction which is not necessarily additive.

**Table 1:** Hallmark gene sets enriched in SB/AKT/c-Met + TAA

Hallmark gene set	SB + TAA vs SB + saline	SB/AKT/c-Met + saline vs SB + saline	SB/AKT/c-Met + TAA vs SB + TAA	SB/AKT/c-Met + TAA vs SB/AKT/c-Met + saline
Apical surface				+ (1.61) (6/37)
Reactive oxygen species pathway				+ (1.45) (9/48)
Up in UV response				+ (1.29) (33/139)
Glycolysis			+ (1.59) (44/185)	+ (1.34) (37/185)
Late estrogen response		+ (1.28) (31/171)	+ (1.76) (32/171)	+ (1.45) (40/171)
IL6/JAK/STAT3 signaling		+ (1.65) (20/81)	+ (1.81) (31/81)	+ (1.27) (32.81)
P53 pathway	+ (1.32) (43/193)	+ (1.25) (54/193)	+ (1.23) (41/193)	+ (1.38) (47/193)
E2F targets	+ (2.55) (107/200)		- (-2.39) (85/200)	+(1.95) (76/200)
Spermatogenesis	+ (1.33) (23/87)		- (-1.15) (19/87)	+ (1.32) (12/87)
G2M checkpoint	+ (2.43) (86/195)	+ (1.36) (55/195)	- (-1.44) (45/195)	+ (2.10) (66/195)
MYC targets (V1)	+ (2.20) (83/199)	- (-1.44) (52/199)	- (-2.53) (84/199)	+ (1.86) (56/199)
MYC targets (V2)	+ (1.68) (22/58)	- (-1.59) (18/58)	- (-1.74) (30/58)	+ (1.81) (23/58)
Adipogenesis	- (-1.89) (61/198)	+ (1.40) (38/198)	+ (1.76) (53/198)	- (-1.57) (30/198)
Fatty acid metabolism	- (-1.76) (48/151)	+ (1.74) (23/151)	+ (2.16) (38/151)	- (-1.45) (30/151)
Bile acid metabolism	- (-2.38) (59/109)	- (-1.86) (37/109)	+ (1.77) (38/109)	- (-1.79) (19/109)

Pathways enriched at false discovery rate < 0.25 level were considered significant. Key: (+), up-regulated in comparison; (-), down-regulated in comparison; (blank), not significantly enriched in comparison; (#), normalized enrichment score; (# / #), number of genes contributing to signal/total number of genes in gene set.

**Table 2:** Hallmark gene sets enriched in SB/AKT/NRas + TAA

Hallmark gene set	SB + TAA vs SB + saline	SB/AKT/NRas + saline vs SB + saline	SB/AKT/NRas + TAA vs SB + TAA	SB/AKT/NRas + TAA vs SB/AKT/NRas + saline
Reactive oxygen species pathway			+ (1.56) (16/48)	+ (1.69) (15/48)
Up in UV response			+ (1.29) (24/139)	+ (1.33) (30/139)
MTORC1 signaling	+ (1.40) (63/199)	+ (1.33) (66/199)	+ (1.51) (37/199)	+ (1.65) (39/199)
P53 pathway	+ (1.32) (43/193)	+ (1.47) (42/193)	+ (1.77) (45/193)	+ (1.39) (56/193)
Glycolysis		+ (1.17) (44/185)	+ (1.77) (50/185)	+ (1.60) (40/185)
Hypoxia		+ (1.67) (67/153)	+ (1.93) (49/183)	+ (1.41) (38/183)
Late estrogen response		+ (1.21) (44/171)	+ (1.72) (41/171)	+ (1.53) (35/171)
Adipogenesis	- (-1.89) (61/198)	- (-1.88) (64/198)	+ (1.93) (55/198)	+ (2.17) (69/198)
Bile acid metabolism	- (-2.38) (59/109)	- (-2.62) (65/109)	+ (1.24) (25/109)	+ (1.41) (27/109)
Fatty acid metabolism	- (-1.76) (48/151)	- (-1.79) (48/151)	+ (2.13) (37/151)	+ (2.08) (46/151)
Heme metabolism	- (-1.61) (55/180)	- (-1.53) (58/180)	+ (1.29) (34/180)	+ (1.48) (39/180)
Xenobiotic metabolism	- (-2.16) (82/193)	- (-2.28) (79/193)	+ (1.59) (44/193)	+ (2.03) (47/193)
G2M checkpoint	+ (2.43) (86/195)	+ (2.12) (95/195)	- (-1.70) (70/195)	- (-1.77) (43/195)
Myogenesis		+ (1.38) (54/175)	- (-1.30) (61/175)	- (-1.25) (34/175)
Epithelial–mesenchymal transition	+ (1.51) (67/179)	+ (1.98) (74/179)	+ (1.26) (38/179)	- (-1.32) (77/179)
TNF alpha signaling via NF-κB	+ (1.41) (65/188)	+ (2.14) (85/188)	+ (1.48) (52/188)	- (-1.66) (50/188)
Angiogenesis		+ (1.57) (15/34)	+ (1.35) (9/34)	- (-1.26) (11/34)
IL6/JAK/STAT3 signaling		+ (2.17) (36/81)	+ (1.70) (33/81)	- (-1.90) (31/81)
Interferon alpha response		+ (2.04) (37/93)	+ (1.44) (42/93)	- (-1.61) (28/93)
MYC targets (V2)	+ (1.68) (22/58)		- (-1.67) (29/58)	+ (1.24) (17/58)

Pathways enriched at false discovery rate < 0.25 level were considered significant. Key: (+), up-regulated in comparison; (-), down-regulated in comparison; (blank), not significantly enriched in comparison; (#), normalized enrichment score; (# / #), number of genes contributing to signal/total number of genes in gene set.

Due to the differences in steatotic phenotype between the groups, lipid metabolism pathway enrichment in the RNA-seq data was further investigated using GSEA with Canonical Pathways gene sets (Supplementary Tables S13 and 14). Both TAA alone and SB/AKT/NRas alone reduced lipid catabolic processes (peroxisomal and mitochondrial beta oxidation), although SB/AKT/NRas alone also increased triglyceride catabolism. Fatty acid synthesis pathways were up-regulated by SB/AKT/c-Met alone, although triglyceride catabolism was also increased and peroxisome activity was decreased. SB/AKT/c-Met + TAA had increased lipid catabolism compared with TAA alone, but little difference from SB/AKT/c-Met alone. SB/AKT/NRas + TAA had increased lipid catabolism compared with TAA alone, but increased synthesis and catabolism compared with SB/AKT/NRas alone. Overall, results largely recapitulated the results from the Hallmark gene sets with further insight into different aspects of lipid metabolism.

## Discussion

In the present study, we comprehensively demonstrate cellular and molecular changes associated with liver cancer development using a combined two-plasmid-HTVI cancer model and a liver injury model which has not been previously used in this context. In human liver cancer, chronic liver injury commonly precedes the development of primary liver cancer and contributes to carcinogenesis through proliferative inflammatory signaling pathways and the development of a permissive cirrhotic microenvironment [5]. However, most studies using plasmid-HTVI models of liver cancer do not incorporate chronic liver injury into their models and thus may not capture this aspect of typical liver cancer biology. In this combined liver cancer-injury model we found liver injury affected a number of pathways. However, although cancer plasmid-TAA combination models developed tumors on a background of fibrosis and inflammation, liver injury did not lead to an increase in tumor size. Thus, the tumor-promoting effect of an injury microenvironment may be context-specific and depends on the genetic drivers of carcinogenesis, the nature of injury and the timeframe of injury.

The use of liver injury agents (primarily chemical or dietary) as tumor promoters to increase tumor number, size and/or incidence is well documented in the context of the carcinogen diethylnitrosamine [19,20,60,61] and some studies using plasmid-HTVI methodology [25–27,29]. Diethylnitrosamine directly and non-specifically damages DNA of proliferating hepatocytes of young mice to cause liver cancer, and liver injury further stimulates proliferation [60]. On the other hand, it is unknown under what circumstances plasmid-HTVI models may be promoted by liver injury, since they are dependent on a well-defined gain-of-function in an oncogenic pathway. Suggested mechanisms for tumor promotion include providing mitogenic stimulus and inducing stemness at the cellular level [62], and inflammatory signaling and cross-talk with stroma at the microenvironmental level [63]. However, in the present study, whilst liver injury did not affect tumor size, it tended to increase tumor numbers. The short time frame of liver injury may mean there was insufficient time for a significant tumor promotion effect to accumulate, although the rapidity of these plasmid-HTVI models limits the time that liver injury can act before mortality due to tumor burden occurs. The transcriptomic signatures of some pathways were cooperatively altered by the plasmids and liver injury, suggesting that there is a degree of synergy. Notably, the enrichment of the p53 pathway in all treatment groups is consistent with a tumor suppressor response to abnormal cell proliferation [64]. On one hand, tumor suppression enhanced by injury may result in multiple slow-growing cancer foci instead of fewer tumors growing rapidly to occupy a larger volume. Alternatively, wild-type p53 can be co-opted in tumor cells to increase tumorigenicity through activating glycolysis [65]. Although synergy was observed in estrogen signalling, its role is unclear at the molecular level as different *in vitro* studies have found it to promote and inhibit HCC growth [66,67]. Given the ambiguity, the exact mechanisms and roles of these altered pathways in tumorigenesis and response to injury require further investigation.

Alternatively, this observation may be interpreted as a differential effect of the injury agent TAA on tumor initiation and promotion depending on biological and/or temporal context. An alternate explanation is that pathways through which plasmids induce cancer and pathways through which TAA-induced injury promotes tumor growth may be different and that there is no appreciable synergistic effect or even an antagonistic effect. Unlike the typical canonical cirrhosis-dysplasia-carcinoma sequence, alternative carcinogenic pathways characterized by different molecular pathways may be active on a non-cirrhotic background [5,30]. Moreover, the plasmid-HTVI model selection may be responsible for this unexpected finding. Matter and colleagues did not find an increase in overall tumor burden using AKT-NRas^G12V^ models combined with 3,5-diethoxycarbonyl-1,4-dihydrocollidine (DDC) [27], although no study to date has combined SB/AKT/c-Met with injury. In addition, differences in mouse strain and age are known to affect susceptibility to liver carcinogens and injury agents [68,69], although this has not been extensively studied in plasmid-HTVI models. Furthermore, using TAA as a liver injury agent, which has a different mechanism of action (toxic metabolite formation targeting lysine and phosphatidylethanolamine) to the more commonly used CCl_4_ (free radical formation) [70,71] may also be partly responsible for current observations. Therefore, more studies are needed to delineate genetic contexts in which different injuries promote or inhibit cancer growth in order to better understand the pathways to carcinogenesis. Some models may be more amenable than others; for example, the AKT/NRas combination may be too aggressive to observe promotion by chronic liver injury.

The use of liver injury agents to convert poorly tumorigenic genetic lesions to fully carcinogenic models is not well characterized compared with combinations with already-carcinogenic models. Chung and colleagues found increased adenoma-to-carcinoma conversion with *SHH* transgene-induced injury [72], while Dauch and colleagues found that CCl_4_ could overcome NRas-induced p19Arf tumor suppressor activity, to increase tumor burden [26]. In the present study, tumorigenesis was not observed in the combination of single oncogene plasmid (NRas, c-Met or AKT) with liver injury, emphasizing the necessity of multiple genetic lesions in rapid tumorigenesis. However, the possibility of tumorigenesis in a single oncogene-injury model over a longer time frame cannot be excluded, particularly the AKT plasmid which is tumorigenic long-term [56]. It should be noted that for long-term studies, TAA can cause liver cancer on a chronic injury-driven liver fibrosis background in some rodent models [73–76], which may mask the effect of a poorly tumorigenic genetic lesion. However, a carefully selected rodent strain and TAA dosing schedule should result in liver injury and fibrosis with minimal carcinogenicity [55].

One of the rationales of developing a liver cancer model with concomitant fibrosis is that cirrhosis is a major risk factor for liver cancer development [6,77]. In the present study, we found that although some TAA-induced changes in expression of fibrosis-related genes, notably *Col1a1*, were observed at this early stage, fibrosis was mild compared with specialized long-term fibrosis models [78]. However, fibrosis occurred in cholangiocellular tumors independent of liver injury. Models of fibrosis development following plasmid-HTVI-initiated cancer [25–27] are useful for investigating how processes involved in liver fibrosis can potentiate carcinogenesis, but may not reflect the clinical scenario of liver cancer developing on a cirrhotic background. The rapidity of many plasmid-HTVI liver cancer models may result in mortality before cirrhosis development, and the induction of fibrosis beforehand can reduce the transfection efficiency of HTVI [79–81]. To overcome these barriers, an appropriate model would be to genetically induce liver cancer on a classic background of liver inflammation and fibrosis. This can be achieved using tamoxifen-inducible Cre-lox recombination [72] in a hepatotoxin-induced fibrotic model. On the other hand, models with mild fibrosis may still be useful as non-cirrhotic liver cancer is well documented, particularly in association with metabolic disturbances where MASLD/MASH directly progresses to HCC [82,83]. Alternatively, since these conditions are characterized primarily by metabolic liver injury, combining a dietary injury with plasmid-HTVI may be of interest.

Inflammation and immune responses are important considerations in liver cancer given its integral role in pathogenesis [6,84]. In the present study, histology and immunostaining showed that TAA increased white blood cell infiltration, although functional information was not gathered. Characterization of inflammation and other pathways was performed primarily by gene expression analysis; increased expression of chemokines and adhesion molecules was associated with TAA in non-cancer groups, consistent with increased white blood cell recruitment. The lack of TAA-induced proinflammatory gene up-regulation in the cancer groups may be explained by the cancer plasmids without TAA which produced minor proinflammatory changes. Alternatively, a basal level of immune response could be due to the foreign DNA introduced by HTVI triggering intracellular pattern recognition receptors such as TLR9, leading to activation of immune responses, although the magnitude of this effect or how long this persists is unknown. This is consistent with the inflammatory pathway GSEA results, and may mask the TAA signal in the cancer plasmids-TAA combinations. Notably, *Lgals3* (encoding galectin-3) is an exception as it is further up-regulated in the cancer plasmids-TAA groups, is up-regulated in a different plasmid-HTVI-injury model [27] and is associated with poorer prognosis in HCC [85]. However, while inflammation is strongly associated with liver cancer, further study to dissect the presence and functions of immune cell subpopulations is necessary as inflammation can be pro- or anti-tumorigenic in different contexts and dependent on the type of inflammation induced [86].

Disturbances in lipid metabolism were noticeable features in these models, which may underscore its importance to some subtypes of liver cancer. Steatosis is a common feature of plasmid-HTVI models activating the PI3K-AKT-mTOR pathway [87–89], with the exception of those with predominantly cholangiocellular tumors [15,90]. Tumorigenesis in these models is strongly (but not completely) dependent on *de novo* lipogenesis by fatty acid synthase [31,91–93]. We found that TAA alone reduced hepatic triglyceride content as previously reported [94] as well as in the non-cancer groups, but not in double-plasmids cancer models. This suggests that the lipolytic effect of TAA may be masked by tumor progression. The differential effect of TAA on lipid metabolism pathways in the different plasmid-HTVI models suggests that its role in cancer is dependent on the genetic background [95]. On the other hand, injury induced by CCl_4_, which is more commonly used to promote fibrosis in liver cancer mouse models, is characterized by steatosis [71,96], although whether this contributes to its ability to promote liver cancer is unknown. This is consistent with lipogenesis being a key feature of early liver tumors [97–99] and being associated with poor patient prognosis [100,101]. For future investigation of the role of lipid metabolism in liver cancer, an alternate experimental approach may involve combining diet-induced metabolic injury with plasmid-HTVI, which may more closely resemble carcinogenesis on a MASLD/MASH background.

There are several limitations that could be addressed in future studies to validate present findings. As the sample size per group is small, increasing the sample size, particularly in the tumor groups, is expected to increase the certainty of the results of this study. Additionally, variability in results could be partly explained due to variation in the transfection efficiency of HTVI. This is unlikely to be due to TAA administration starting one week after HTVI because SB transposase activity following plasmid injection occurs over the first four days [102]. Mouse age at time of HTVI may also be an additional factor, although whether this is mediated by transfection efficiency or differing biological response to the plasmid is unknown. The use of a reporter such as luciferase would be useful to monitor *in vivo* transfection efficiency [102].

## Conclusion

In the present study, liver injury with TAA was combined with plasmid-HTVI liver cancer models to produce a fibrotic and proinflammatory microenvironment for cancer development as this is more relevant to the development of human liver cancer. TAA administration was associated with inflammation and fibrosis, increased tumor density in combination with SB/AKT/NRas, but did not appreciably increase tumor size. We conclude that plasmid-HTVI-TAA is able to generate cancer with an injury microenvironment and alters its presentation. However, liver injury may have different effects on cancer development depending on the genetic drivers and active oncogenic pathways. Thus, the choice of plasmid-HTVI model and injury agent may influence the extent to which injury promotes cancer development.

## Clinical perspectives

- Plasmid-HTVI models of liver cancer may not model cancer development on a background of liver injury and fibrosis, which is common in human liver cancer. This study sought to characterize cancer development in plasmid-HTVI models combined with liver injury.
- Despite thioacetamide causing liver injury and fibrosis, the combination of plasmid-HTVI and thioacetamide did not significantly increase tumor size, but increased multiplicity of small neoplastic lesions compared with plasmid-HTVI without injury. Cancer and/or liver injury up-regulated profibrotic and proinflammatory genes while metabolic pathway genes were mostly down-regulated.
- Combining liver injury and plasmid-HTVI models of liver cancer can improve our understanding of how liver injury contributes to the development of liver cancer with different genetic drivers. This may enable identification of potential therapeutic targets which would not be present in liver cancer models without injury.

## Supplementary Material

Supplementary Figures S1-S15 and Tables S1-S14

## Data Availability

The RNA-seq dataset can be found on the NCBI Gene Expression Omnibus database (accession ID 174074). The other datasets generated during and/or analysed during the current study are available from the corresponding author on reasonable request.

## Open Access

Open access for this article was enabled by the participation of University Of Melbourne in an all-inclusive *Read & Publish* agreement with Portland Press and the Biochemical Society under a transformative agreement with CAUL.

## Abbreviations

ALP: alkaline phosphatase
ALT: alanine aminotransferase
AST: aspartate aminotransferase
BSA: bovine serum albumin
CCl_4_: carbon tetrachloride
CD: cluster of differentiation
cDNA: complementary DNA
CK: cytokeratin
DAB: 3,3′-diaminobenzidine
DAPI: 4’,6-diamidino-2-phenylindole
DDC: 3,5-diethoxycarbonyl-1,4-dihydrocollidine
dNTPs: deoxyribonucleotide triphosphates
FDR: false discovery rate
FFPE: formalin-fixed paraffin embedded
GFP: green fluorescent protein
GSEA: gene set enrichment analysis
H&E: hematoxylin and eosin
HCC: hepatocellular carcinoma
HRP: horseradish peroxidase
HTVI: hydrodynamic tail vein injection
i.p.: intraperitoneal
log2FC: log2 fold change
MASH: metabolic dysfunction-associated steatohepatitis
MASLD: metabolic dysfunction-associated steatotic liver disease
OCT: optimal cutting temperature medium
PBS: phosphate-buffered saline
PI3K: phosphatidylinositol-3-kinase
PSR: picro-sirius red
RNA-seq: RNA sequencing
SB: sleeping beauty
TAA: thioacetamide
TG: triglyceride
TRITC: tetramethylrhodamine
UMI: unique molecular identifier
α-SMA: alpha smooth muscle actin
