# Supplementary material for: Oncogenic plasmid DNA and liver injury agent dictates liver cancer development in a mouse model
Source: Clin Sci (Lond). 2024 Sep 26;138(19):1227–48. doi: 10.1042/CS20240560 (PMC11427747; doi:10.1042/CS20240560)
Supplement: Supplementary Figures S1-S15 and Tables S1-S14 [file CS-2024-0560_supp.pdf]

Oncogenic plasmid DNA and liver injury agent dictates liver cancer development in a mouse model

Vincent Chiu[1,2], Christine Yee[1,2], Nathan Main[1,2], Igor Stevanovski[1,2], Matthew Watt[3], Trevor Wilson[4], Peter Angus[5], Tara Roberts[1,6], Nicholas Shackel[1,2], Chandana Herath*[1,2,7]

Author affiliations:

[1]Ingham Institute for Applied Medical Research, Liverpool, New South Wales, Australia. [2]South Western Sydney Clinical School, UNSW Sydney, Liverpool, New South Wales, Australia. [3]School of Biomedical Sciences, University of Melbourne, Victoria, Australia. [4]Hudson Institute of Medical Research, Monash University, Victoria, Australia. [5]Department of Gastroenterology and Hepatology, Austin Health, Heidelberg, Victoria, Australia [6]School of Medicine, Western Sydney University, Campbelltown, New South Wales, Australia. [7]Department of Medicine, Austin Health, University of Melbourne, Victoria, Australia.

Corresponding author: Chandana Herath, Department of Medicine, The University of Melbourne, Austin Health, Heidelberg, Victoria 3084, Australia; email: cherath@unimelb.edu.au

Supplementary Table 1: OpenArray Fibrosis gene panel

Gene symbol	Gene name	Taqman Assay ID
Acta2	Alpha smooth muscle actin	Mm00725412_s1
Bsg	Basigin	Mm01144228_g1
Col1a1	Collagen type 1 alpha 1	Mm00801666_g1
Col3a1	Collagen type 3 alpha 1	Mm01254476_m1
Col4a1	Collagen type 4 alpha 1	Mm01210125_m1
Ctgf	Connective tissue growth factor	Mm01192932_g1
Hgf	Hepatocyte growth factor	Mm01135193_m1
Mmp2	Matrix metalloproteinase 2	Mm00439498_m1
Mmp3	Matrix metalloproteinase 3	Mm00440295_m1
Hif1a	Hypoxia inducible factor alpha	Mm00468869_m1
Nos2	Inducible nitric oxide synthase	Mm00440502_m1
Mmp9	Matrix metalloproteinase 9	Mm00442991_m1
Il1b	Interleukin-1beta	Mm00434226_m1
Mmp13	Matrix metalloproteinase 13	Mm00439491_m1
Mmp14	Matrix metalloproteinase 14	Mm00485054_m1
Pdgfb	Platelet derived growth factor beta	Mm00440677_m1
Timp1	Tissue inhibitor of matrix metalloproteinase 1	Mm00441818_m1
Timp2	Tissue inhibitor of matrix metalloproteinase 2	Mm00441825_m1
Vegfa	Vascular endothelial growth factor alpha	Mm01281449_m1
Kdr	Vascular endothelial growth factor receptor 2	Mm01222421_m1
Fn1	Fibronectin	Mm0125744_m1
Alb	Albumin	Mm00802090_m1
Pecam1	Platelet endothelial cell adhesion molecule	Mm01242576_m1
Cd163	Cluster of differentiation 163	Mm00474091_m1
Ubc	Ubiquitin C	Mm02525934_g1
Hprt	Hypoxanthine-guanine phosphoribosyltransferase	Mm01545399_m1
Rplp0	60S acidic ribosomal protein P0	Mm00725448_s1
Aldob	Aldolase B	Mm00523293_m1

Supplementary Table 2: OpenArray Immune gene panel

Gene symbol	Gene name	Taqman Assay ID
Ccl2	C-C motif chemokine ligand 2	Mm00441242_m1
Ccl11	C-C motif chemokine ligand 11	Mm00441238_m1
Ccr3	C-C chemokine receptor 3	Mm00515543_s1
Cxcr3	C-X-C motif chemokine receptor 3	Mm99999054_s1
Cxcl10	C-X-C motif chemokine ligand 10	Mm00445235_m1
Fas	Fas receptor	Mm01204974_m1
Icam1	Intracellular adhesion molecule 1	Mm00516023_m1
Ifng	Interferon gamma	Mm01168134_m1
Il6	Interleukin-6	Mm00446190_m1
Il10	Interleukin-10	Mm00439614_m1
Itgal	Integrin subunit alpha L or CD11a	Mm00801807_m1
Itgb5	Integrin beta 5	Mm00439825_m1
Lgals3	Galectin-3	Mm00802901_m1
Ngf	Nerve growth factor	Mm00443039_m1
Sdc1	Syndecan-1	Mm00448920_g1
Slc16a1	Monocarboxylate transporter 1	Mm01306379_m1
Slc16a3	Monocarboxylate transporter 1	Mm00446102_m1
Slc3a2	4F2 cell-surface antigen heavy chain	Mm00500521_m1
Tgfb1	Transforming growth factor beta 1	Mm01178820_m1
Tnf	Tumour necrosis factor	Mm00443258_m1
Tnfrsf1a	TNF receptor 1	Mm00441883_g1
Nfkb1	Nuclear factor of kappa light polypeptide gene enhancer in B cells 1	Mm00476361_m1
Tlr4	Toll-like recepor 4	Mm00445273_m1
Ccl5	C-C motif chemokine ligand 5	Mm01302427_m1
Ubc	Ubiquitin C	Mm02525934_g1
Hprt1	Hypoxanthine-guanine phosphoribosyltransferase	Mm01545399_m1
Rplp0	60S acidic ribosomal protein P0	Mm00725448_s1
Aldob	Aldolase B	Mm00523293_m1

Supplementary Table 3: Genes differentially expressed in all single treatment vs SB + saline comparisons. All genes were upregulated in both comparisons except those marked (*) (downregulated in both comparisons).

Ensembl Gene ID	Gene Symbol	SB + TAA	SB/AKT/c-Met + saline	SB/AKT/NRas + saline
ENSMUSG00000004038	Gstm3	7.27	11.00	9.31
ENSMUSG00000015962	1700016C15Rik	11.21	15.39	11.36
ENSMUSG00000021208	Ifi27l2b	4.13	6.01	7.50
ENSMUSG00000027712	Anxa5	2.93	4.92	4.94
ENSMUSG00000029254	Stap1	9.91	8.88	5.32
ENSMUSG00000030004	Nat8*	0.37	0.28	0.21
ENSMUSG00000030047	Arhgap25	5.92	7.69	5.02
ENSMUSG00000031150	Ccdc120	5.25	9.44	9.57
ENSMUSG00000032080	Apoa4	4.04	19.55	8.44
ENSMUSG00000034634	Ly6d	14.71	32.18	47.34
ENSMUSG00000040562	Gstm2	2.39	2.78	3.09
ENSMUSG00000052684	Jun	2.25	3.20	2.91
ENSMUSG00000055254	Ntrk2	9.66	10.57	11.36
ENSMUSG00000074802	Gas2l3	3.50	3.18	2.72

Supplementary Table 4: Genes differentially expressed in both SB/AKT/c-Met + saline and SB/AKT/NRas + saline vs SB + saline and their fold change relative to SB + saline. All genes were upregulated in both comparisons except those marked (*) (downregulated in both comparisons).

Ensembl Gene ID	Gene Symbol	SB/AKT/c-Met + saline	SB/AKT/NRas + saline
ENSMUSG00000002944	Cd36	3.12	2.95
ENSMUSG00000017167	Cntnap1	11.61	8.18
ENSMUSG00000017405	Nek8	5.41	5.10
ENSMUSG00000020312	Shc2	9.63	10.57
ENSMUSG00000022003	Slc25a30	5.01	5.97
ENSMUSG00000022364	Tbc1d31	3.66	2.42
ENSMUSG00000024109	Nrxn1	5.75	3.04
ENSMUSG00000024403	Atp6v1g2	9.22	6.12
ENSMUSG00000024526	Cidea	99.74	31.91
ENSMUSG00000027068	Dhrs9	7.22	5.62
ENSMUSG00000028664	Ephb2	13.19	5.64
ENSMUSG00000030278	Cidec	17.72	8.04
ENSMUSG00000030827	Fgf21	12.19	9.42
ENSMUSG00000031271	Serpina7	4.73	4.41
ENSMUSG00000035184	Fam124a	5.12	3.95
ENSMUSG00000038641	Akr1d1*	0.42	0.32
ENSMUSG00000041959	S100a10	2.79	2.35
ENSMUSG00000042041	2010003K11Rik	8.54	5.37
ENSMUSG00000045136	Tubb2b	24.72	20.15
ENSMUSG00000053168	9030619P08Rik	4.39	2.58
ENSMUSG00000058672	Tubb2a	6.49	4.77
ENSMUSG00000069170	Adgrv1	6.39	3.72
ENSMUSG00000074373	Gm10680	17.80	8.77
ENSMUSG00000074639	Rdh16f2*	0.35	0.29
ENSMUSG00000111977	AC132265.1	19.61	25.24

Supplementary Table 5: Differentially expressed genes in SB/AKT/c-Met + TAA and not differentially expressed in SB/AKT/c-Met + saline or SB + TAA vs SB + saline comparisons. Key: (+), upregulated in comparison; (-), downregulated in comparison; FDR, false discovery rate.

Ensembl Gene ID	Gene symbol	FDR	Direction of change
**SB/AKT/c-Met + TAA vs SB + TAA**			
ENSMUSG00000061397	Krt79	7.25×10^{-5}	+
ENSMUSG00000021957	Tkt	0.00188	+
ENSMUSG00000032420	Nt5e	0.00281	+
ENSMUSG00000040660	Cyp2b9	0.00287	+
ENSMUSG00000079042	Apela	0.00299	+
ENSMUSG00000114242	AC154355.1	0.00526	+
ENSMUSG00000005547	Cyp2a5	0.00544	+
ENSMUSG00000075044	Slc22a29	0.00568	+
ENSMUSG00000044071	Fam19a2	0.00647	+
ENSMUSG00000022824	Muc13	0.0105	+
ENSMUSG00000046324	Ermp1	0.0137	+
ENSMUSG00000018459	Slc13a3	0.0150	+
ENSMUSG00000061540	Orm2	0.0165	+
ENSMUSG00000067656	Slc22a27	0.0195	+
ENSMUSG00000022947	Cbr3	0.0257	+
ENSMUSG00000049109	Themis	0.0257	+
ENSMUSG00000030739	Myh14	0.0442	+
ENSMUSG00000039131	Gipc2	0.0442	+
ENSMUSG00000004951	Hspb1	0.0457	+
**SB/AKT/c-Met + TAA vs SB/AKT/c-Met + saline**			
ENSMUSG00000061099	Gapdhs	6.59×10^{-4}	+
ENSMUSG00000047026	Acsm4	0.00743	+
ENSMUSG00000084403	Rps15a-ps8	0.00884	+
ENSMUSG00000043681	Fam25c	0.00972	+
ENSMUSG00000111877	Gm6477	0.0155	+
ENSMUSG00000006494	Pdk1	0.0156	-
ENSMUSG00000057933	Gsta2	0.0281	+
ENSMUSG00000001918	Slc1a5	0.0385	+
ENSMUSG00000037437	Adam32	0.0386	+
ENSMUSG00000110099	Gm45344	0.0401	-
ENSMUSG00000021573	Tppp	0.0409	-
ENSMUSG00000018411	Mapt	0.0490	-
ENSMUSG00000073460	Pnldc1	0.0490	-
ENSMUSG00000092203	1110038B12Rik	0.0490	+
ENSMUSG00000105031	Gm3511	0.0497	+

Supplementary Table 6: Differentially expressed genes in both SB/AKT/c-Met + TAA vs SB/AKT/c-Met + saline and SB/AKT/c-Met + TAA vs SB + TAA comparisons. Key: (+), upregulated in comparison; (-), downregulated in comparison; (*), also downregulated in SB + TAA.

Ensembl Gene ID	Gene symbol	SB/AKT/c-Met + TAA vs SB/AKT/c-Met + saline	SB/AKT/c-Met + TAA vs SB + TAA
ENSMUSG00000040808	S100g	+	+
ENSMUSG00000032849	Abcc4	+	+
ENSMUSG00000025037	Maoa	+	+
ENSMUSG00000031561	Tenm3*	-	+
ENSMUSG00000112636	AC152414.2	+	+
ENSMUSG00000074489	Bglap3	+	+
ENSMUSG00000022215	Fitm1*	-	+
ENSMUSG00000074254	Cyp2a4	+	+
ENSMUSG00000038327	Serpinb9f	+	+

Supplementary Table 7: Differentially expressed genes in SB/AKT/NRas + TAA and not differentially expressed in SB/AKT/NRas + saline or SB + TAA vs SB + saline comparisons. Key: (+), upregulated in comparison; (-), downregulated in comparison; FDR, false discovery rate.

Ensembl Gene ID	Gene symbol	FDR	Direction of change
**SB/AKT/NRas + TAA vs SB + TAA**			
ENSMUSG00000021957	Tkt	5.74×10^{-6}	+
ENSMUSG00000022824	Muc13	8.81×10^{-5}	+
ENSMUSG00000012187	Mogat1	9.06×10^{-5}	+
ENSMUSG00000112636	AC152414.2	1.95×10^{-4}	+
ENSMUSG00000075044	Slc22a29	4.30×10^{-4}	+
ENSMUSG00000040660	Cyp2b9	6.62×10^{-4}	+
ENSMUSG00000018459	Slc13a3	7.00×10^{-4}	+
ENSMUSG00000110841	Gpx4-ps2	7.00×10^{-4}	+
ENSMUSG00000067656	Slc22a27	0.00101	+
ENSMUSG00000001891	Ugp2	0.00146	+
ENSMUSG00000030739	Myh14	0.00161	+
ENSMUSG00000037419	Endod1	0.00161	+
ENSMUSG00000046324	Ermp1	0.00187	+
ENSMUSG00000016024	Lbp	0.00284	+
ENSMUSG00000029188	Slc34a2	0.00284	+
ENSMUSG00000044071	Fam19a2	0.00284	+
ENSMUSG00000032418	Me1	0.00285	+
ENSMUSG00000003721	Insig2	0.00289	+
ENSMUSG00000033147	Slc22a15	0.00347	+
ENSMUSG00000072944	Nup62cl	0.00673	+
ENSMUSG00000108415	Gm30146	0.00844	+
ENSMUSG00000040875	Osbpl10	0.00871	+
ENSMUSG00000026413	Pkp1	0.00876	+
ENSMUSG00000023057	Fabp2	0.00937	+
ENSMUSG00000063873	Slc24a3	0.0103	+
ENSMUSG00000033208	S100b	0.0122	+
ENSMUSG00000020142	Slc1a4	0.0126	+
ENSMUSG00000022951	Rcan1	0.0131	+
ENSMUSG00000031995	St14	0.0131	+
ENSMUSG00000114242	AC154355.1	0.0132	+
ENSMUSG00000024143	Rhoq	0.0137	+
ENSMUSG00000050914	Ankrd37	0.0146	+
ENSMUSG00000030545	Pex11a	0.0151	+
ENSMUSG00000027890	Gstm4	0.0165	+
ENSMUSG00000041237	Pklr	0.0165	+
ENSMUSG00000028532	Cachd1	0.0172	+

Supplementary Table 7 (continued)

Ensembl Gene ID	Gene symbol	FDR	Direction of change
SB/AKT/NRas + TAA vs SB + TAA			
ENSMUSG00000028755	Cda	0.0174	+
ENSMUSG00000115143	AC142114.1	0.0188	+
ENSMUSG00000063354	Slc39a4	0.0206	+
ENSMUSG00000056185	Snx32	0.0206	+
ENSMUSG00000085936	2610307P16Rik	0.0206	+
ENSMUSG00000061762	Tac1	0.0208	-
ENSMUSG00000109841	E330011O21Rik	0.0208	+
ENSMUSG00000060981	Hist1h4h	0.0228	+
ENSMUSG00000045912	C2cd4c	0.0254	+
ENSMUSG00000004951	Hspb1	0.0262	+
ENSMUSG00000030165	Klrd1	0.0262	-
ENSMUSG00000022096	Hr	0.0272	+
ENSMUSG00000051483	Cbr1	0.0272	+
ENSMUSG00000016458	Wt1	0.0278	-
ENSMUSG00000024131	Slc3a1	0.0278	-
ENSMUSG00000060317	Acnat2	0.0278	+
ENSMUSG00000047492	Inhbe	0.0281	+
ENSMUSG00000055653	Gpc3	0.0288	+
ENSMUSG00000028359	Orm3	0.0289	+
ENSMUSG00000041550	Serpina5	0.0305	+
ENSMUSG00000026854	Usp20	0.0323	+
ENSMUSG00000004383	Large1	0.0328	+
ENSMUSG00000031934	Panx1	0.0341	+
ENSMUSG00000090555	Gm8893	0.0342	-
ENSMUSG00000028803	Nipal3	0.0349	+
ENSMUSG00000021943	Gdf10	0.0354	-
ENSMUSG00000062342	Serpinb9e	0.0354	+
ENSMUSG00000047026	Acsm4	0.0365	+
ENSMUSG00000097451	Rian	0.0386	+
ENSMUSG00000078719	Msmp	0.0422	-
ENSMUSG00000109825	Gm45589	0.0464	+
ENSMUSG00000033685	Ucp2	0.0479	+
ENSMUSG00000038327	Serpinb9f	0.0497	+

Supplementary Table 7 (continued)

Ensembl Gene ID	Gene symbol	FDR	Direction of change
SB/AKT/NRas + TAA vs SB/AKT/NRas + saline			
ENSMUSG00000060550	H2-Q7	0.00389	-
ENSMUSG00000043681	Fam25c	0.00431	+
ENSMUSG00000057933	Gsta2	0.00431	+
ENSMUSG00000026688	Mgst3	0.00567	+
ENSMUSG00000064307	Lrrc51	0.0219	+
ENSMUSG00000106829	Gm3786	0.0225	-
ENSMUSG00000025190	Got1	0.0258	+
ENSMUSG00000051314	Ffar2	0.0307	-
ENSMUSG00000001918	Slc1a5	0.0440	+

Supplementary Table 8: Differentially expressed genes in both SB/AKT/NRas + TAA vs SB/AKT/NRas + saline and SB/AKT/NRas + TAA vs SB + TAA comparisons. Key: (+), upregulated in comparison; (*), Also upregulated in SB/AKT/NRas + saline vs SB + saline comparison.

Ensembl Gene ID	Gene symbol	SB/AKT/NRas + TAA vs SB + TAA	SB/AKT/NRas + TAA vs SB/AKT/NRas + saline
ENSMUSG00000036111	Lmo1	+	+
ENSMUSG00000032420	Nt5e	+	+
ENSMUSG00000076617	Ighm	+	+
ENSMUSG00000053168	9030619P08Rik*	+	+
ENSMUSG00000074254	Cyp2a4*	+	+
ENSMUSG00000025037	Maoa	+	+
ENSMUSG00000059668	Krt4	+	+
ENSMUSG00000074489	Bglap3	+	+
ENSMUSG00000032849	Abcc4*	+	+
ENSMUSG00000041220	Elovl6	+	+
ENSMUSG00000039131	Gipc2*	+	+
ENSMUSG00000091867	Cyp2a22	+	+
ENSMUSG00000109814	Gm45847	+	+
ENSMUSG00000050737	Ptges	+	+
ENSMUSG00000005547	Cyp2a5	+	+
ENSMUSG00000022947	Cbr3*	+	+

Supplementary Table 9: Enriched gene ontology terms in differentially expressed genes on SB + TAA vs SB + saline comparison

GO: Biological process	
**Upregulated in SB + TAA**	**Downregulated in SB + TAA**
glutathione metabolic process	lipid storage
regeneration	regulation of lipid storage
response to stilbenoid	negative regulation of lipid storage
response to stimulus	circadian behavior
establishment of chromosome localization	locomotor rhythm
chromosome organization	mitochondrion morphogenesis
chromosome segregation	drug metabolic process
regulation of chromosome segregation	cellular response to xenobiotic stimulus
regulation of mitotic metaphase/anaphase transition	xenobiotic metabolic process
regulation of mitotic sister chromatid separation	xenobiotic catabolic process
regulation of mitotic sister chromatid segregation	taurine metabolic process
negative regulation of mitotic sister chromatid segregation	benzene-containing compound metabolic process
regulation of sister chromatid segregation	organic hydroxy compound metabolic process
negative regulation of sister chromatid segregation	negative regulation of cellular carbohydrate metabolic process
nuclear chromosome segregation	negative regulation of carbohydrate metabolic process
mitotic spindle assembly checkpoint	regulation of gluconeogenesis
negative regulation of mitotic metaphase/anaphase transition	negative regulation of gluconeogenesis
mitotic sister chromatid segregation	catabolic process
negative regulation of chromosome segregation	exogenous drug catabolic process
regulation of attachment of spindle microtubules to kinetochore	drug catabolic process
metaphase/anaphase transition of mitotic cell cycle	alkanesulfonate metabolic process
mitotic spindle checkpoint	response to xenobiotic stimulus
sister chromatid segregation	fatty acid derivative metabolic process
regulation of metaphase/anaphase transition of cell cycle	response to drug
negative regulation of metaphase/anaphase transition of cell cycle	heat generation
negative regulation of mitotic sister chromatid separation	negative regulation of lipid metabolic process
cytokinesis	negative regulation of lipid biosynthetic process
cellular response to DNA damage stimulus	oxidation-reduction process
cell division	insulin receptor signaling pathway

Supplementary Table 9 (continued)

GO: Biological process	
**Upregulated in SB + TAA**	**Downregulated in SB + TAA**
microtubule-based process	linoleic acid metabolic process
cell cycle	organic acid metabolic process
xenobiotic metabolic process	lipid metabolic process
cellular response to xenobiotic stimulus	negative regulation of insulin secretion involved in cellular response to glucose stimulus
response to xenobiotic stimulus	small molecule metabolic process
cellular response to stimulus	icosanoid metabolic process
response to toxic substance	omega-hydroxylase P450 pathway
protein localization to kinetochore	arachidonic acid metabolic process
protein localization to chromosome, centromeric region	epoxygenase P450 pathway
DNA conformation change	steroid metabolic process
DNA packaging	very long-chain fatty acid metabolic process
chromosome condensation	steroid biosynthetic process
spindle assembly checkpoint	bile acid metabolic process
mitotic cell cycle checkpoint	small molecule catabolic process
mitotic DNA integrity checkpoint	monocarboxylic acid catabolic process
spindle checkpoint	alpha-amino acid catabolic process
G2 DNA damage checkpoint	lipid catabolic process
DNA integrity checkpoint	organic acid catabolic process
microtubule cytoskeleton organization	carboxylic acid catabolic process
spindle organization	fatty acid catabolic process
organelle fission	cellular amino acid catabolic process
nuclear division	cellular amino acid metabolic process
negative regulation of mitotic nuclear division	monocarboxylic acid metabolic process
regulation of mitotic nuclear division	oxoacid metabolic process
microtubule cytoskeleton organization involved in mitosis	carboxylic acid metabolic process
negative regulation of nuclear division	fatty acid metabolic process
regulation of nuclear division	cellular catabolic process
cytoskeleton organization	lipid biosynthetic process
G2/M transition of mitotic cell cycle	small molecule biosynthetic process
regulation of cell cycle phase transition	cellular lipid metabolic process
negative regulation of cell cycle phase transition	long-chain fatty acid metabolic process
negative regulation of G2/M transition of mitotic cell cycle	unsaturated fatty acid metabolic process
regulation of mitotic cell cycle phase transition	organic acid biosynthetic process
negative regulation of mitotic cell cycle phase transition	rhythmic behavior

Supplementary Table 9 (continued)

GO: Biological process	
**Upregulated in SB + TAA**	**Downregulated in SB + TAA**
mitotic G2 DNA damage checkpoint	circadian rhythm
mitotic cell cycle phase transition	alpha-amino acid metabolic process
cell cycle G2/M phase transition	
regulation of G2/M transition of mitotic cell cycle	
mitotic G2/M transition checkpoint	
regulation of cell cycle G2/M phase transition	
meiotic cell cycle process	
meiotic chromosome segregation	
spindle midzone assembly	
mitotic spindle assembly	
spindle assembly	
mitotic spindle organization	
mitotic cytokinesis	
cytoskeleton-dependent cytokinesis	
mitotic cell cycle	
regulation of cell cycle	
cell cycle process	
spindle elongation	
mitotic chromosome condensation	
chromosome localization	
positive regulation of cell cycle	
regulation of mitotic cell cycle	
mitotic cell cycle process	
negative regulation of cell cycle	
regulation of cell cycle process	
positive regulation of mitotic cell cycle	
positive regulation of cell cycle process	
positive regulation of exit from mitosis	
positive regulation of cell cycle phase transition	
positive regulation of mitotic cell cycle phase transition	
cell cycle phase transition	
mitotic spindle midzone assembly	
mitotic spindle elongation	
cell cycle checkpoint	
negative regulation of cell cycle process	
negative regulation of mitotic cell cycle	
attachment of spindle microtubules to kinetochore	

Supplementary Table 9 (continued)

GO: Cellular component	
**Upregulated in SB + TAA**	**Downregulated in SB + TAA**
extracellular region	extracellular region
midbody	extracellular space
intercellular bridge	peroxisome
supramolecular complex	microbody
supramolecular fiber	
supramolecular polymer	
polymeric cytoskeletal fiber	
microtubule	
serine/threonine protein kinase complex	
cyclin-dependent protein kinase holoenzyme complex	
protein kinase complex	
chromosome	
non-membrane-bounded organelle	
interstitial matrix	
condensed chromosome	
spindle	
microtubule organizing center	
centrosome	
condensed nuclear chromosome kinetochore	
kinetochore	
condensed chromosome kinetochore	
condensed nuclear chromosome, centromeric region	
condensed chromosome, centromeric region	
condensed nuclear chromosome	
condensed chromosome outer kinetochore	
mitotic spindle	
spindle pole	
spindle microtubule	
intracellular non-membrane-bounded organelle	
microtubule cytoskeleton	
basement membrane	
cytoskeleton	
chromosomal region	
chromosome, centromeric region	
nuclear chromosome	

Supplementary Table 9 (continued)

GO: Molecular function	
**Upregulated in SB + TAA**	**Downregulated in SB + TAA**
glutathione transferase activity	catalytic activity
	sterol esterase activity
	odorant binding
	protein-hormone receptor activity
	molecular transducer activity
	pheromone binding
	heme binding
	iron ion binding
	oxidoreductase activity
	tetrapyrrole binding
	monooxygenase activity
	insulin-activated receptor activity
	signaling receptor activity
	steroid hydroxylase activity
	arachidonic acid monooxygenase activity
	aromatase activity
	arachidonic acid epoxygenase activity
	oxidoreductase activity, acting on paired donors, with incorporation or reduction of molecular oxygen, reduced flavin or flavoprotein as one donor, and incorporation of one atom of oxygen
	oxidoreductase activity, acting on paired donors, with incorporation or reduction of molecular oxygen
	triglyceride lipase activity

Supplementary Table 10: Hallmark gene sets enriched in all single treatment groups compared to SB + saline. Gene set names have been renamed for readability. Key: (+), upregulated in comparison; (-), downregulated in comparison.

Hallmark gene set	SB + TAA vs SB + saline	SB/AKT/c-Met + saline vs SB + saline	SB/AKT/NRas + saline vs SB + saline
Allograft rejection	+	+	+
Apoptosis	+	+	+
DNA repair	+	+	+
Epithelial-mesenchymal transition	+	+	+
G2M checkpoint	+	+	+
Inflammatory response	+	+	+
Interferon gamma response	+	+	+
Up in KRAS signalling	+	+	+
Mitotic spindle	+	+	+
MTORC1 signalling	+	+	+
P53 pathway	+	+	+
TNF alpha signalling via NF-κB	+	+	+
Complement	-	+	+
Early estrogen response	-	+	+
Adipogenesis	-	+	-
Fatty acid metabolism	-	+	-
Peroxisome	-	+	-
Bile acid metabolism	-	-	-
Coagulation	-	-	-
Down in KRAS signalling	-	-	-
Oxidative phosphorylation	-	-	-
Wnt/β-catenin signalling	-	-	-
Xenobiotic metabolism	-	-	-

Supplementary Table 11: Enriched gene ontology terms in differentially expressed genes on SB/AKT/NRas + saline or SB/AKT/c-Met + saline vs SB + saline comparison

GO: Biological process	
**Upregulated in SB/AKT/NRas + saline**	**Downregulated in SB/AKT/NRas + saline**
signaling	metabolic process
response to stilbenoid	protein homotetramerization
regulation of fibroblast proliferation	drug metabolic process
response to stimulus	cellular response to xenobiotic stimulus
establishment of chromosome localization	xenobiotic metabolic process
organelle fission	nitrogen cycle metabolic process
chromosome segregation	hormone metabolic process
cell communication	C21-steroid hormone metabolic process
regulation of transferase activity	cellular hormone metabolic process
regulation of macrophage cytokine production	organic hydroxy compound catabolic process
positive regulation of macrophage cytokine production	fatty acid derivative metabolic process
regulation of chromosome segregation	sulfur compound metabolic process
regulation of mitotic metaphase/anaphase transition	benzene-containing compound metabolic process
regulation of mitotic sister chromatid separation	catabolic process
regulation of mitotic sister chromatid segregation	indole-containing compound metabolic process
regulation of sister chromatid segregation	biosynthetic process
nuclear chromosome segregation	response to xenobiotic stimulus
mitotic sister chromatid segregation	response to drug
negative regulation of chromosome segregation	one-carbon metabolic process
regulation of attachment of spindle microtubules to kinetochore	organic cyclic compound metabolic process
sister chromatid segregation	organic substance biosynthetic process
regulation of metaphase/anaphase transition of cell cycle	steroid metabolic process
negative regulation of metaphase/anaphase transition of cell cycle	lipid catabolic process
regulation of cell division	cellular lipid metabolic process
regulation of biological quality	small molecule catabolic process
cell division	lipid biosynthetic process
microtubule-based process	pyruvate biosynthetic process
regulation of developmental process	pyridine-containing compound biosynthetic process

Supplementary Table 11 (continued)

GO: Biological process	
**Upregulated in SB/AKT/NRas + saline**	**Downregulated in SB/AKT/NRas + saline**
mitotic cell cycle	nucleoside phosphate biosynthetic process
regulation of cell cycle	nicotinamide nucleotide metabolic process
cell cycle process	nucleotide metabolic process
response to chemical	nucleotide biosynthetic process
regulation of multicellular organismal process	nucleoside phosphate metabolic process
cell cycle	NAD biosynthetic process
positive regulation of biological process	pyridine nucleotide biosynthetic process
positive regulation of phagocytosis, engulfment	pyridine nucleotide metabolic process
positive regulation of membrane invagination	nicotinamide nucleotide biosynthetic process
response to metal ion	pteridine-containing compound metabolic process
cellular response to metal ion	exogenous drug catabolic process
cellular response to inorganic substance	drug catabolic process
cellular response to calcium ion	response to organic cyclic compound
response to calcium ion	ribonucleoside bisphosphate metabolic process
regulation of localization	purine nucleoside bisphosphate metabolic process
regulation of cellular component organization	pyrimidine-containing compound metabolic process
chromosome condensation	beta-alanine metabolic process
positive regulation of multicellular organismal process	cellular modified amino acid catabolic process
response to organic substance	urea cycle
cellular response to stimulus	urea metabolic process
signal transduction	sulfur compound catabolic process
microtubule cytoskeleton organization	cellular amine metabolic process
spindle organization	cellular biogenic amine metabolic process
nuclear division	tryptophan metabolic process
negative regulation of mitotic nuclear division	indolalkylamine metabolic process
regulation of mitotic nuclear division	aromatic amino acid family catabolic process
microtubule cytoskeleton organization involved in mitosis	oxidation-reduction process
negative regulation of nuclear division	amine metabolic process
regulation of nuclear division	cellular modified amino acid metabolic process
cytoskeleton organization	response to glucocorticoid

Supplementary Table 11 (continued)

GO: Biological process	
**Upregulated in SB/AKT/NRas + saline**	**Downregulated in SB/AKT/NRas + saline**
cellular response to chemical stimulus	response to corticosteroid
positive regulation of cell proliferation	dicarboxylic acid metabolic process
regulation of cell proliferation	organic cyclic compound catabolic process
G2/M transition of mitotic cell cycle	lipid metabolic process
regulation of cell cycle phase transition	nucleoside bisphosphate metabolic process
regulation of mitotic cell cycle phase transition	purine-containing compound metabolic process
mitotic cell cycle phase transition	tetrahydrofolate metabolic process
cell cycle G2/M phase transition	folic acid-containing compound metabolic process
spindle midzone assembly	nucleobase metabolic process
mitotic spindle assembly	small molecule metabolic process
spindle assembly	L-phenylalanine metabolic process
mitotic spindle organization	erythrose 4-phosphate/phosphoenolpyruvate family amino acid catabolic process
positive regulation of transferase activity	L-phenylalanine catabolic process
regulation of protein kinase activity	heterocycle catabolic process
regulation of protein serine/threonine kinase activity	organonitrogen compound catabolic process
regulation of kinase activity	steroid biosynthetic process
cell cycle checkpoint	icosanoid metabolic process
negative regulation of cell cycle process	arachidonic acid metabolic process
negative regulation of mitotic cell cycle	epoxygenase P450 pathway
spindle localization	long-chain fatty acid metabolic process
positive regulation of mitotic cell cycle	unsaturated fatty acid metabolic process
positive regulation of cell cycle process	sulfur amino acid catabolic process
mitotic cytokinesis	serine family amino acid catabolic process
cytoskeleton-dependent cytokinesis	ornithine metabolic process
regulation of cytokinesis	erythrose 4-phosphate/phosphoenolpyruvate family amino acid metabolic process
positive regulation of cytokinesis	cellular nitrogen compound catabolic process
spindle elongation	kynurenine metabolic process
chromosome localization	pyrimidine-containing compound catabolic process
cell cycle phase transition	nucleobase catabolic process
mitotic spindle midzone assembly	thymine catabolic process
mitotic spindle elongation	pyrimidine nucleobase catabolic process
positive regulation of cell cycle	pyrimidine nucleobase metabolic process
regulation of mitotic cell cycle	thymine metabolic process

Supplementary Table 11 (continued)

GO: Biological process	
**Upregulated in SB/AKT/NRas + saline**	**Downregulated in SB/AKT/NRas + saline**
mitotic cell cycle process	aromatic amino acid family metabolic process
negative regulation of cell cycle	aromatic compound catabolic process
regulation of cell cycle process	small molecule biosynthetic process
spindle assembly checkpoint	arginine metabolic process
negative regulation of cell cycle phase transition	glutamate metabolic process
negative regulation of mitotic sister chromatid segregation	arginine biosynthetic process
negative regulation of sister chromatid segregation	glutamine family amino acid biosynthetic process
negative regulation of mitotic cell cycle phase transition	alpha-amino acid biosynthetic process
mitotic cell cycle checkpoint	organic cyclic compound biosynthetic process
mitotic spindle assembly checkpoint	organic acid metabolic process
negative regulation of mitotic metaphase/anaphase transition	monocarboxylic acid metabolic process
spindle checkpoint	monocarboxylic acid catabolic process
mitotic spindle checkpoint	monocarboxylic acid biosynthetic process
negative regulation of mitotic sister chromatid separation	cellular amino acid metabolic process
cytokinesis	organic acid biosynthetic process
	organic acid catabolic process
	oxoacid metabolic process
	carboxylic acid catabolic process
	carboxylic acid biosynthetic process
	carboxylic acid metabolic process
	fatty acid metabolic process
	aspartate family amino acid catabolic process
	cellular amino acid catabolic process
	alpha-amino acid catabolic process
	cellular amino acid biosynthetic process
	sulfur amino acid metabolic process
	organonitrogen compound biosynthetic process
	nucleobase-containing small molecule metabolic process
	serine family amino acid metabolic process
	aspartate family amino acid metabolic process
	glutamine family amino acid metabolic process
	alpha-amino acid metabolic process
	organic substance catabolic process
	cellular catabolic process

Supplementary Table 11 (continued)

GO: Cellular component	
**Upregulated in SB/AKT/NRas + saline**	**Downregulated in SB/AKT/NRas + saline**
extracellular region	mitochondrial membrane
extracellular space	mitochondrion
midbody	
supramolecular complex	
supramolecular fiber	
supramolecular polymer	
polymeric cytoskeletal fiber	
microtubule	
centralspindlin complex	
cell surface	
plasma membrane	
condensed chromosome	
cytoskeleton	
spindle	
condensed chromosome outer kinetochore	
kinetochore	
condensed chromosome kinetochore	
condensed nuclear chromosome, centromeric region	
condensed chromosome, centromeric region	
mitotic spindle	
spindle pole	
microtubule cytoskeleton	
condensed nuclear chromosome outer kinetochore	
chromosome, centromeric region	

Supplementary Table 11 (continued)

GO: Molecular function	
**Upregulated in SB/AKT/NRas + saline**	**Downregulated in SB/AKT/NRas + saline**
protein kinase binding	catalytic activity
protein binding	transaminase activity
kinase binding	heme binding
	vitamin binding
	iron ion binding
	hydrolase activity, acting on ether bonds
	lyase activity
	oxidoreductase activity, acting on the CH-NH group of donors
	pyridoxal phosphate binding
	tetrapyrrole binding
	oxidoreductase activity
	transferase activity, transferring nitrogenous groups
	lipase activity
	carboxylic acid binding
	amino acid binding
	transition metal ion binding
	organic acid binding
	vitamin B6 binding
	sterol esterase activity
	steroid hydroxylase activity
	aromatase activity
	arachidonic acid monooxygenase activity
	arachidonic acid epoxygenase activity
	oxidoreductase activity, acting on paired donors, with incorporation or reduction of molecular oxygen, NAD(P)H as one donor, and incorporation of one atom of oxygen
	oxidoreductase activity, acting on paired donors, with incorporation or reduction of molecular oxygen, reduced flavin or flavoprotein as one donor, and incorporation of one atom of oxygen
	oxidoreductase activity, acting on single donors with incorporation of molecular oxygen, incorporation of two atoms of oxygen

Supplementary Table 11 (continued)

GO: Molecular function	
**Upregulated in SB/AKT/NRas + saline**	**Downregulated in SB/AKT/NRas + saline**
	oxidoreductase activity, acting on single donors with incorporation of molecular oxygen
	steroid dehydrogenase activity
	monooxygenase activity
	methyl indole-3-acetate esterase activity
	oxidoreductase activity, acting on the CH-OH group of donors, NAD or NADP as acceptor
	steroid dehydrogenase activity, acting on the CH-OH group of donors, NAD or NADP as acceptor
	3-beta-hydroxy-delta5-steroid dehydrogenase activity
	oxidoreductase activity, acting on CH-OH group of donors
	oxidoreductase activity, acting on paired donors, with incorporation or reduction of molecular oxygen
	triglyceride lipase activity
**GO: Biological process**	
**Upregulated in SB/AKT/c-Met + saline**	**Downregulated in SB/AKT/c-Met + saline**
response to stilbenoid	

Supplementary Table 12: Hallmark gene sets enriched in both SB/AKT/c-Met + saline and SB/AKT/NRas + saline groups compared to SB + saline. Gene set names have been renamed for readability. Pathways enriched at false discovery rate < 0.25 level were considered significant. Key: (+), upregulated in comparison; (#), normalised enrichment score; (# / #), number of genes contributing to signal / total number of genes in gene set.

Hallmark gene set	SB/AKT/c-Met + saline vs SB + saline	SB/AKT/NRas + saline vs SB + saline
Androgen response	+ (1.47) (25/95)	+ (1.26) (20/95)
Cholesterol homeostasis	+ (1.40) (18/73)	+ (1.58) (21/73)
Late estrogen response	+ (1.28) (31/171)	+ (1.21) (44/171)
Hypoxia	+ (1.47) (28/153)	+ (1.67) (67/153)
IL2/STAT5 signalling	+ (1.38) (54/182)	+ (1.53) (75/182)
IL6/JAK/STAT3 signalling	+ (1.65) (20/81)	+ (2.17) (36/81)
Interferon alpha response	+ (1.60) (40/93)	+ (2.04) (37/93)
Myogenesis	+ (1.45) (37/175)	+ (1.38) (54/175)
Protein secretion	+ (1.35) (26/94)	+ (1.38) (35/94)
Down in UV response	+ (1.20) (30/142)	+ (1.27) (30/142)

Supplementary Table 13: Canonical Pathway gene sets related to lipid metabolism enriched in SB/AKT/c-Met + TAA. Pathways enriched at false discovery rate < 0.25 level were considered significant. Key: (+), upregulated in comparison; (-), downregulated in comparison; (blank), not significantly enriched in comparison; (#), normalised enrichment score; (# / #), number of genes contributing to signal / total number of genes in gene set.

Canonical Pathway gene set	SB + TAA vs SB + saline	SB/AKT/c-Met + saline vs SB + saline	SB/AKT/c-Met + TAA vs SB + TAA	SB/AKT/c-Met + TAA vs SB/AKT/c-Met + saline
Reactome peroxisomal lipid metabolism	- (-2.32) (19/28)		+ (1.97) (13/28)	
Reactome peroxisomal protein import	- (-2.31) (35/61)			
KEGG peroxisome	- (-2.29) (40/76)	- (-1.65) (25/76)		
Reactome mitochondrial fatty acid beta oxidation	- (-2.00) (18/35)			
Reactome fatty acid metabolism	- (-1.92) (57/157)		+ (1.88) (47/157)	
KEGG fatty acid metabolism	- (-1.78) (19/39)		+ (1.83) (9/39)	+ (1.62) (6/39)
Reactome triglyceride metabolism		+ (1.98) (11/30)		
Reactome triglyceride catabolism		+ (1.96) (9/19)		
Reactome fatty acyl CoA biosynthesis		+ (1.84) (9/30)		
KEGG biosynthesis of unsaturated fatty acids		+ (1.83) (5/21)		

Supplementary Table 14: Canonical Pathway gene sets related to lipid metabolism enriched in SB/AKT/NRas + TAA. Pathways enriched at false discovery rate < 0.25 level were considered significant. Key: (+), upregulated in comparison; (-), downregulated in comparison; (blank), not significantly enriched in comparison; (#), normalised enrichment score; (# / #), number of genes contributing to signal / total number of genes in gene set.

Canonical Pathway gene set	SB + TAA vs SB + saline	SB/AKT/NRas + saline vs SB + saline	SB/AKT/NRas + TAA vs SB + TAA	SB/AKT/NRas + TAA vs SB/AKT/NRas + saline
Reactome peroxisomal lipid metabolism	- (-2.32) (19/28)	- (-2.07) (16/28)	+ (1.81) (10/28)	+ (1.55) (9/28)
Reactome peroxisomal protein import	- (-2.31) (35/61)	- (-2.19) (40/61)		+ (1.55) (27/61)
KEGG peroxisome	- (-2.29) (40/76)	- (-2.39) (41/76)		+ (1.68) (29/76)
Reactome mitochondrial fatty acid beta oxidation	- (-2.00) (18/35)	- (-1.81) (22/35)		+ (1.83) (15/35)
Reactome fatty acid metabolism	- (-1.92) (57/157)	- (-1.81) (52/157)	+ (1.90) (52/157)	+ (2.13) (44/157)
KEGG fatty acid metabolism	- (-1.78) (19/39)	- (-2.15) (25/39)		+ (1.93) (11/39)
Reactome triglyceride metabolism		+ (1.33) (12/30)	+ (1.86) (11/30)	
Reactome triglyceride catabolism		+ (1.31) (7/19)	+ (1.70) (7/19)	
KEGG biosynthesis of unsaturated fatty acids			+ (1.64) (13/21)	
Reactome synthesis of very long chain fatty acyl CoAs				+ (1.84) (2/20)
Reactome fatty acyl CoA biosynthesis				+ (1.62) (4/30)

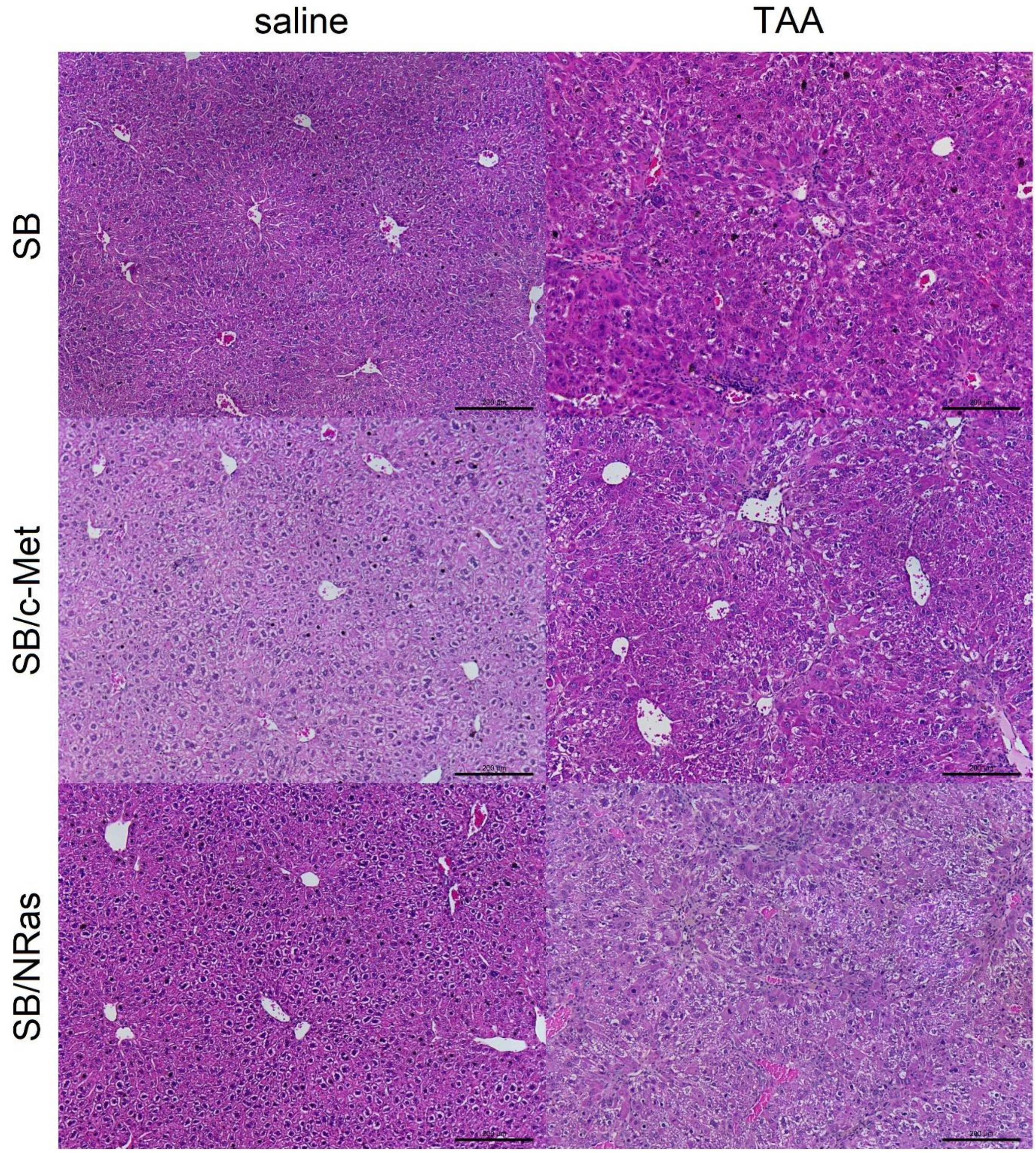

Supplementary Figure 1: Histopathology of SB/c-Met and SB/NRas was similar to SB-alone. Representative haematoxylin and eosin sections from SB alone, SB/c-Met and SB/NRas with and without TAA. Scale bar = 200 µm. TAA, thioacetamide.

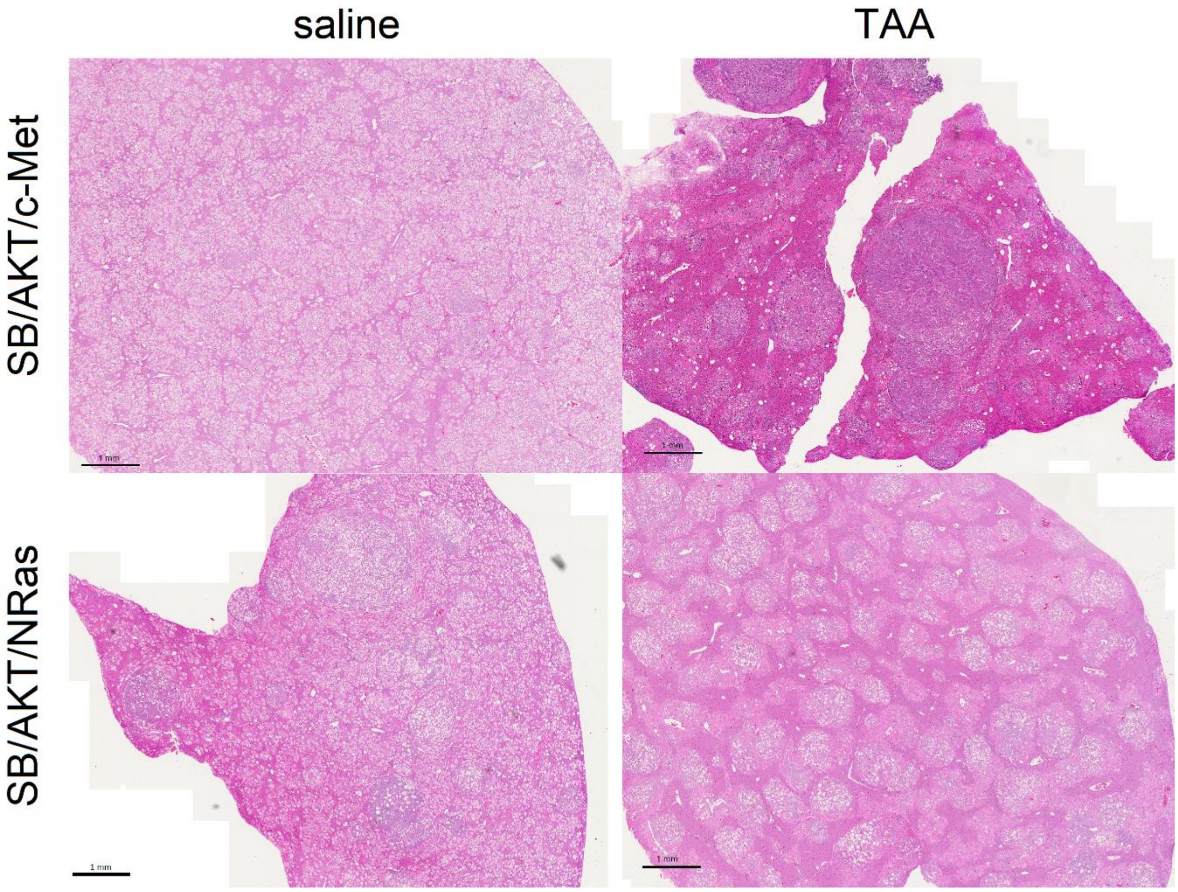

Supplementary Figure 2: Low power fields of view from whole-section scans of livers. Representative haematoxylin and eosin sections from SB/AKT/c-Met and SB/AKT/NRas with and without TAA. Scale bar = 1 mm. TAA, thioacetamide.

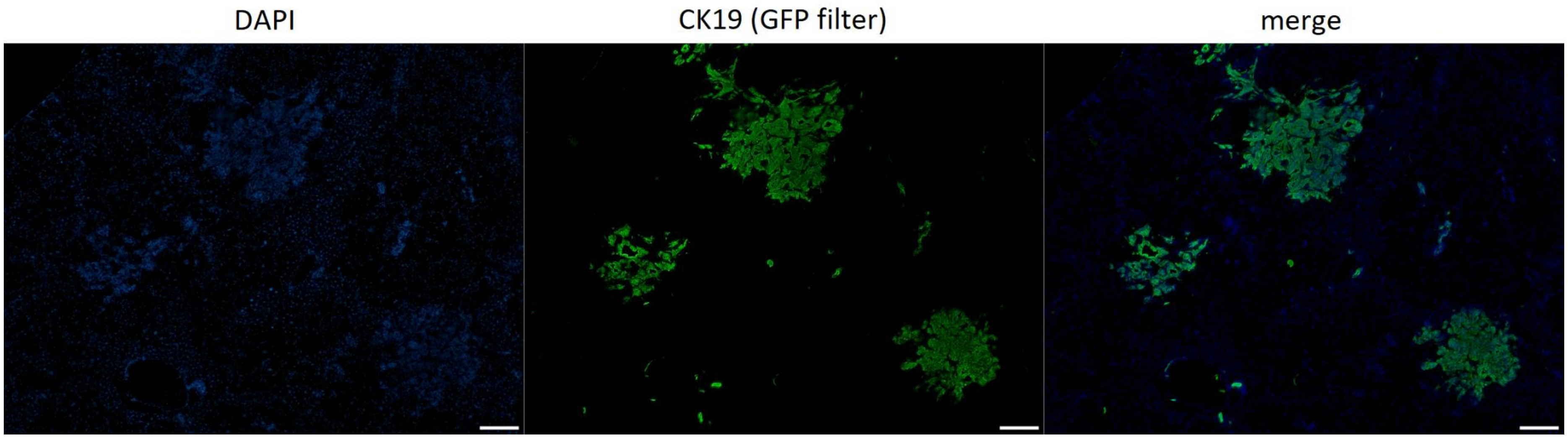

Supplementary Figure 3: Some tumours in SB/AKT/NRas groups were positive for cytokeratin 19 by immunofluorescent stain. Representative section from SB/AKT/NRas + TAA. Scale bar = 200 µm. CK19, cytokeratin 19; DAPI, 4’,6-diamidino-2-phenylindole (blue filter); GFP, green fluorescent protein (green filter); TAA, thioacetamide.

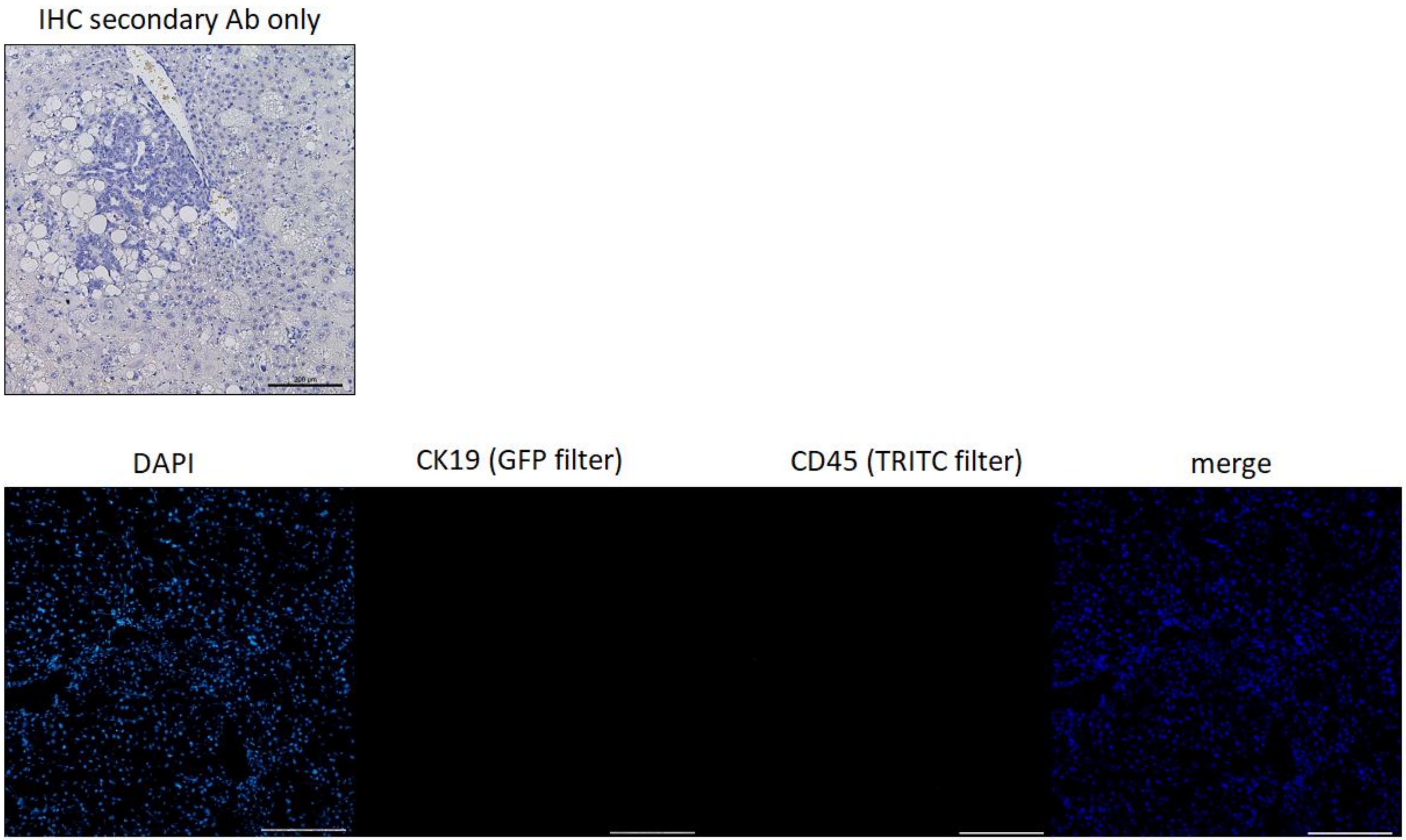

Supplementary Figure 4: Secondary antibody-only control sections for immunohistochemistry and immunofluorescence. Scale bar = 200 µm. CK19, cytokeratin 19; CD45, cluster of differentiation 45; DAPI, 4’,6-diamidino-2-phenylindole (blue filter); GFP, green fluorescent protein (green filter); TRITC, tetramethylrhodamine (red filter).

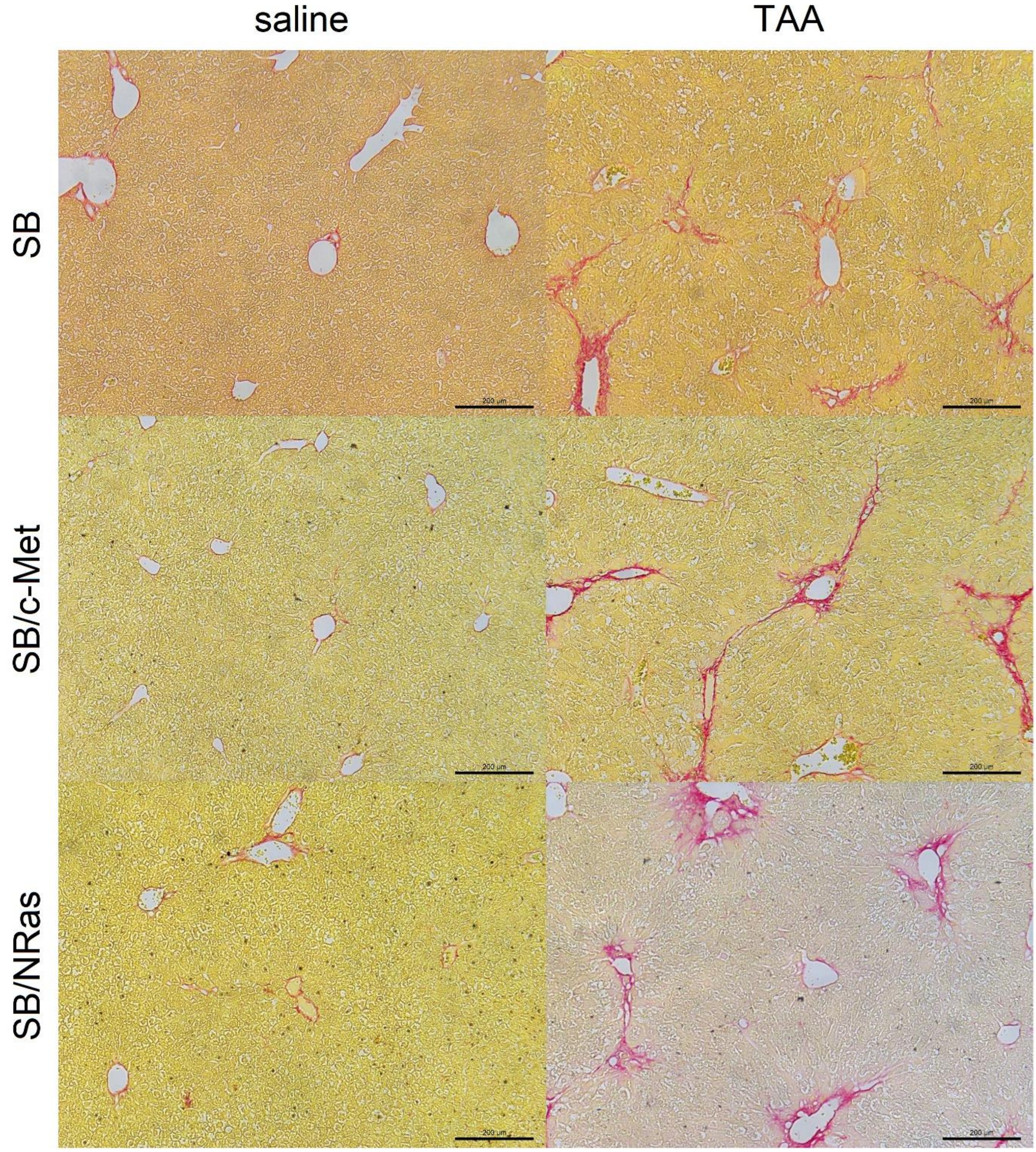

Supplementary Figure 5: Collagen staining of SB/c-Met and SB/NRas was similar to SB-alone. Representative Picro-sirius red sections from SB alone, SB/c-Met and SB/NRas with and without TAA. Scale bar = 200 µm. TAA, thioacetamide.

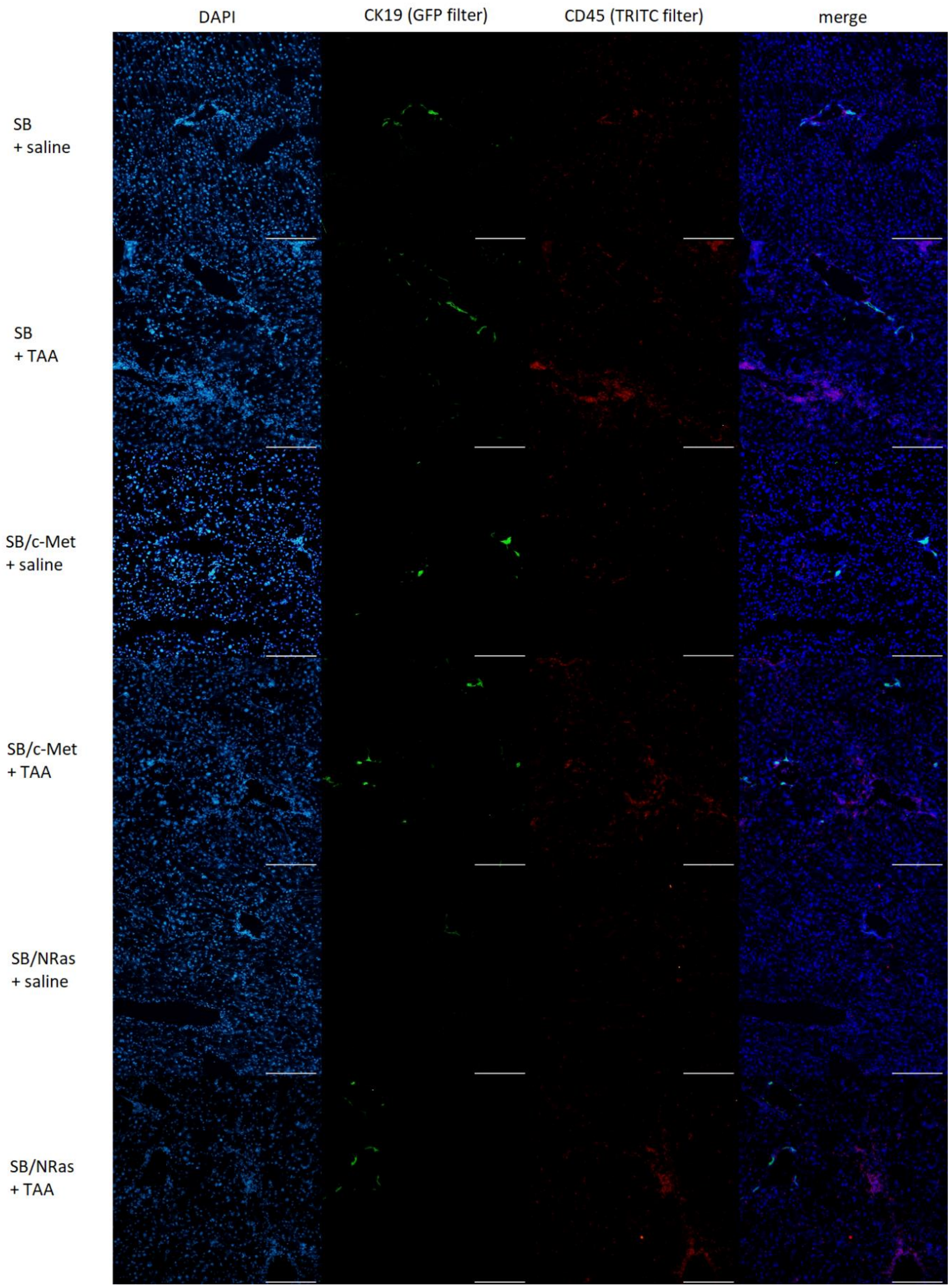

Supplementary Figure 6 (legend on next page)

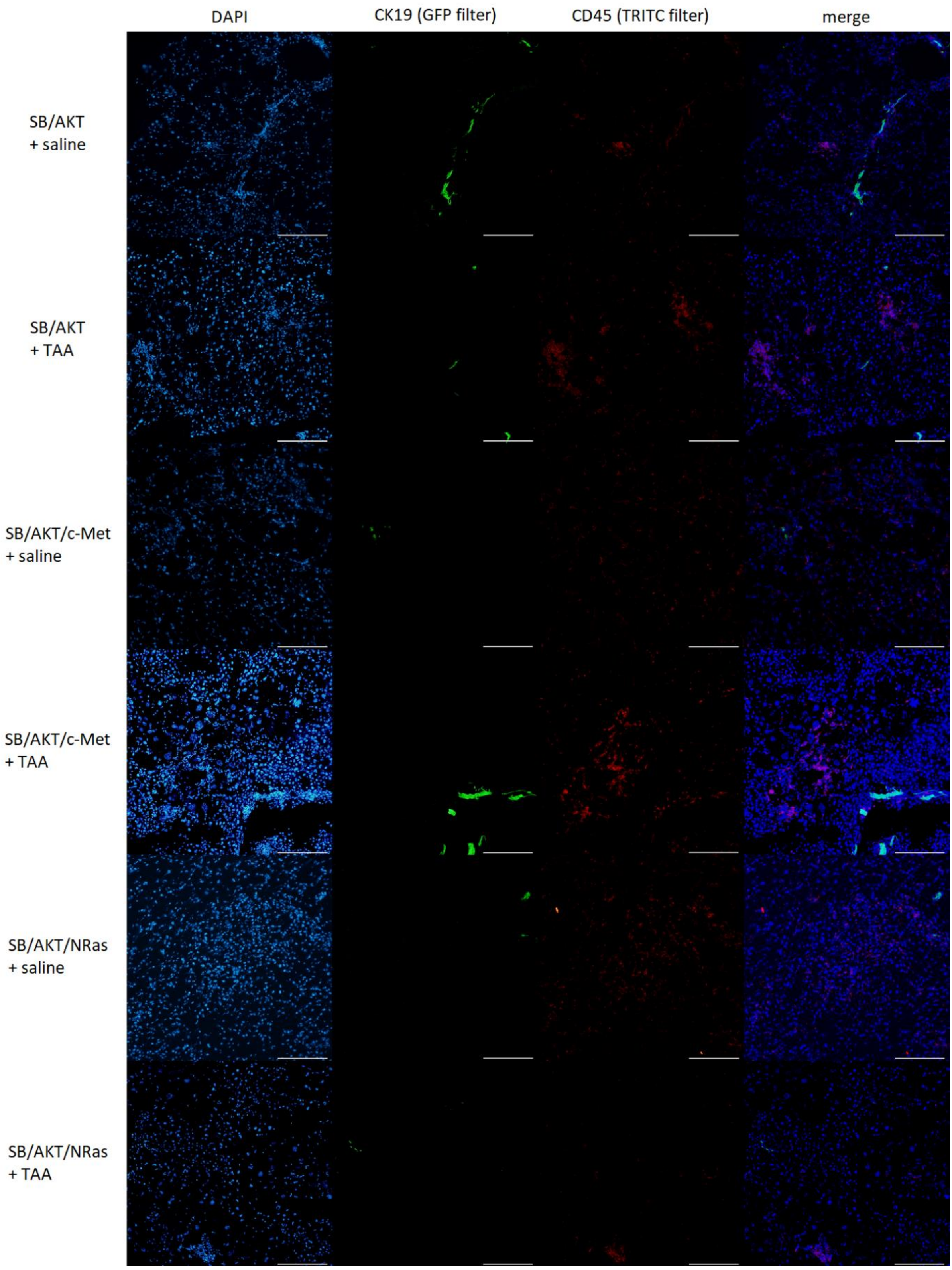

Supplementary Figure 6: Cytokeratin 19 and cluster of differentiation 45 immunofluorescent staining of mouse livers, representative non-tumour fields of view. Cytokeratin 19 was imaged with a green filter, cluster of differentiation 45 was imaged with a red filter, nuclear stain (Hoechst 33342) was imaged with blue filter. Scale bar = 200 µm.

CK19, cytokeratin 19; CD45, cluster of differentiation 45; DAPI, 4’,6-diamidino-2-phenylindole (blue filter); GFP, green fluorescent protein (green filter); TAA, thioacetamide; TRITC, tetramethylrhodamine (red filter).

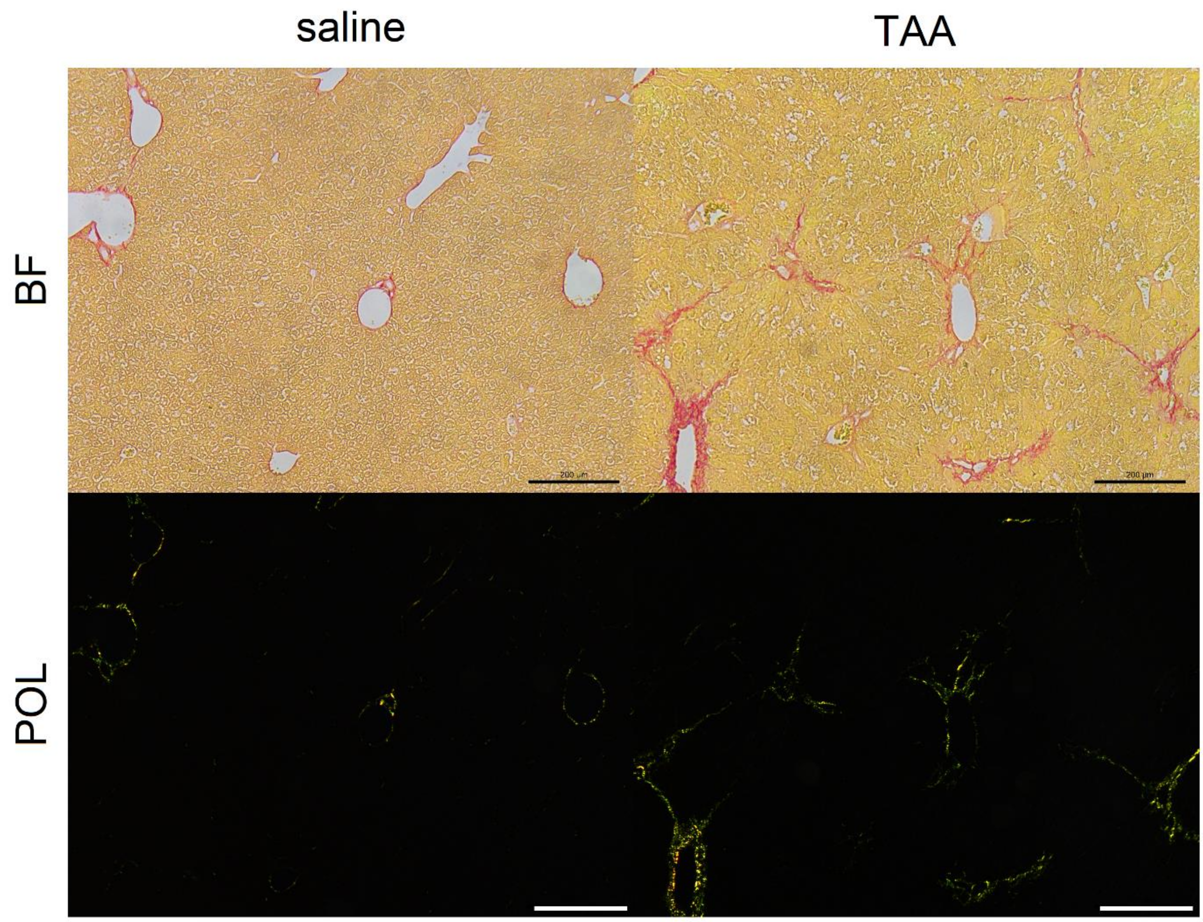

Supplementary Figure 7: Polarized light microscopy confirmed the specific staining of collagen by Picro-sirius red. Representative field of view of SB + saline and SB + TAA, paired bright field and polarized light micrograph. Scale bar = 200 µm. BF, bright field; POL, polarized light micrograph of same field of view; TAA, thioacetamide.

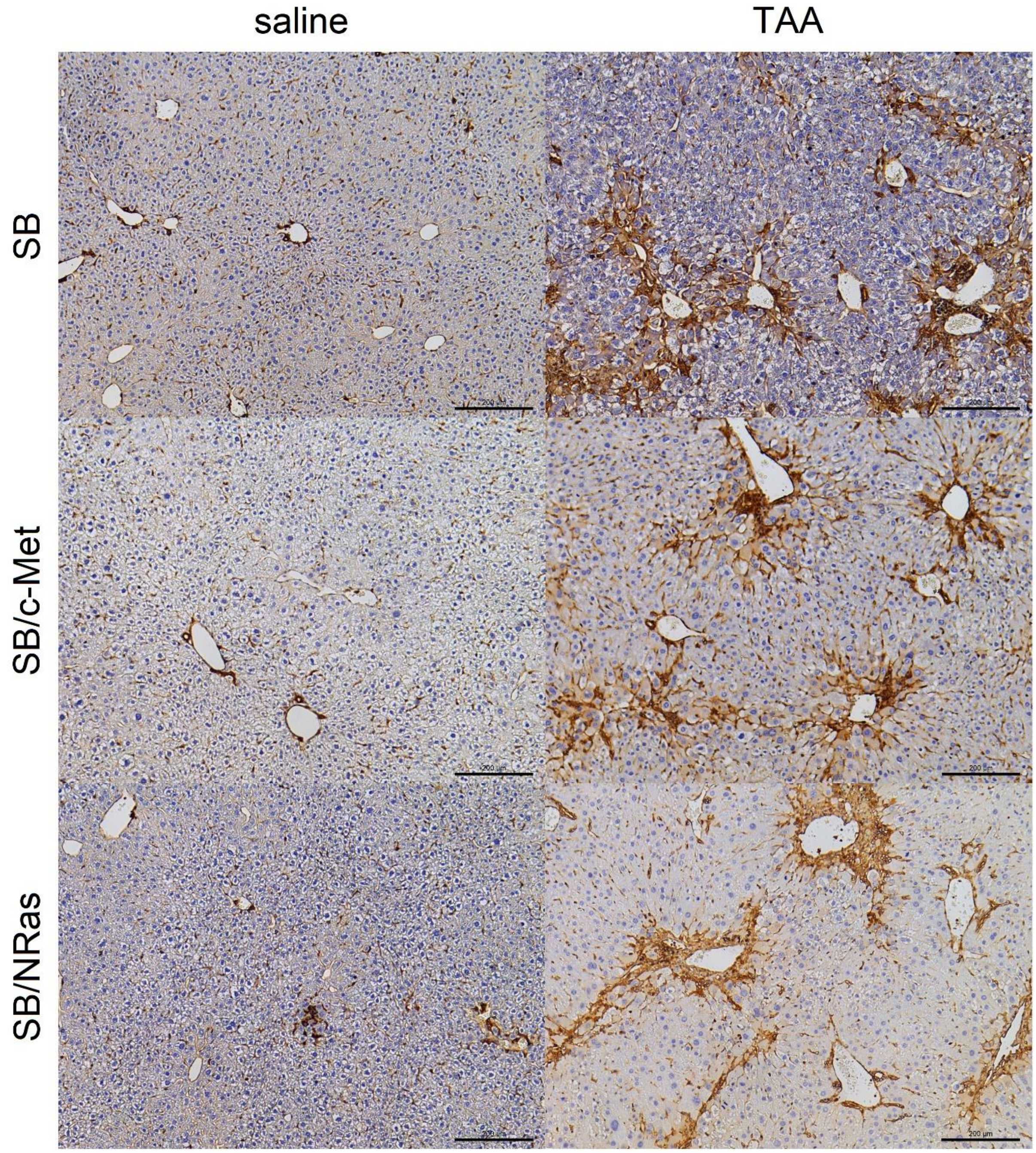

Supplementary Figure 8: Alpha smooth muscle actin staining of SB/c-Met and SB/NRas was similar to SB-alone. Representative alpha smooth muscle actin immunohistochemistry sections from SB alone, SB/c-Met and SB/NRas with and without TAA. Scale bar = 200 µm. TAA, thioacetamide.

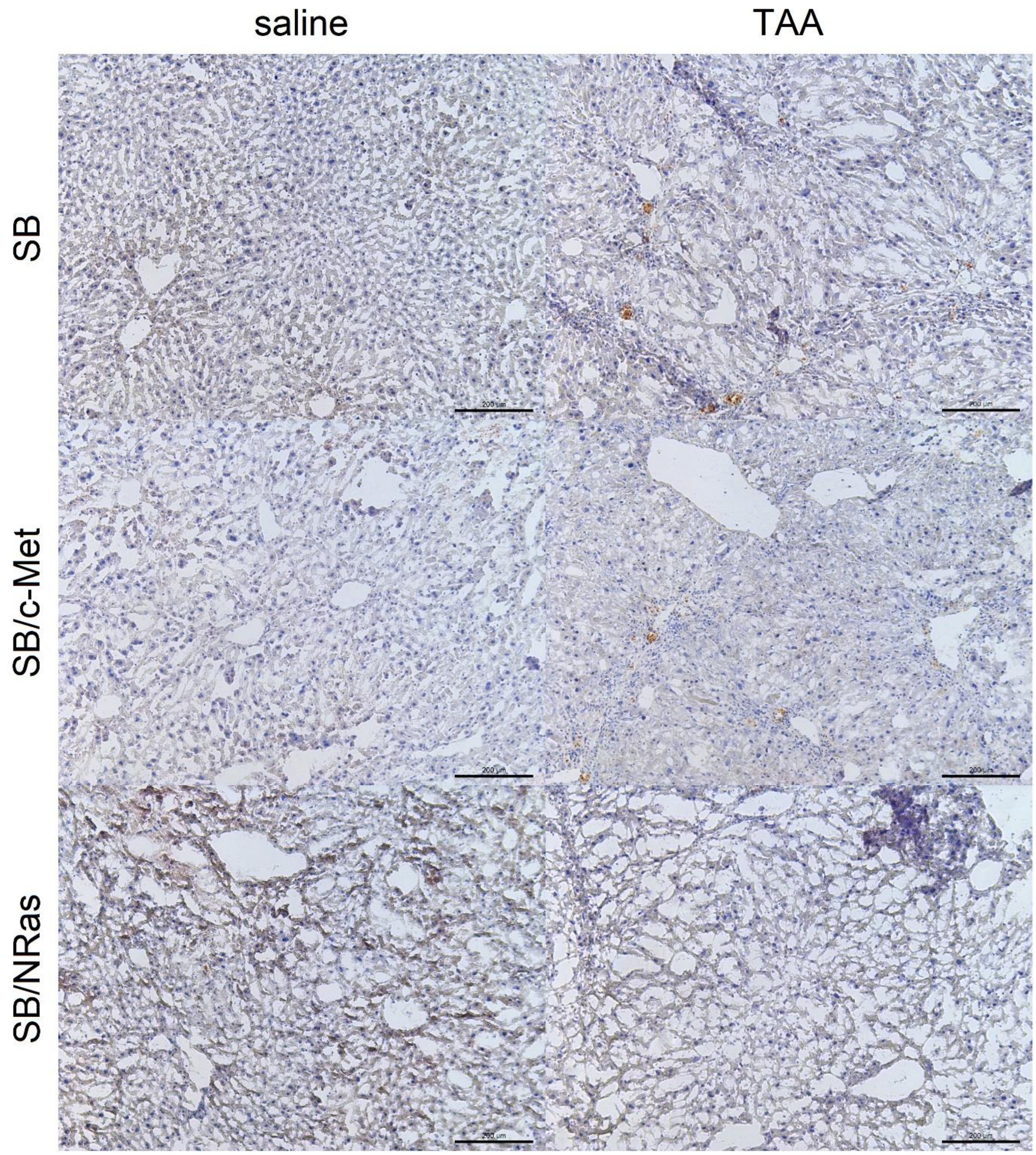

Supplementary Figure 9: Oil Red O staining of SB/c-Met and SB/NRas was similar to SB-alone. Representative Oil Red O sections from SB alone, SB/c-Met and SB/NRas with and without TAA. Scale bar = 200 µm. TAA, thioacetamide.

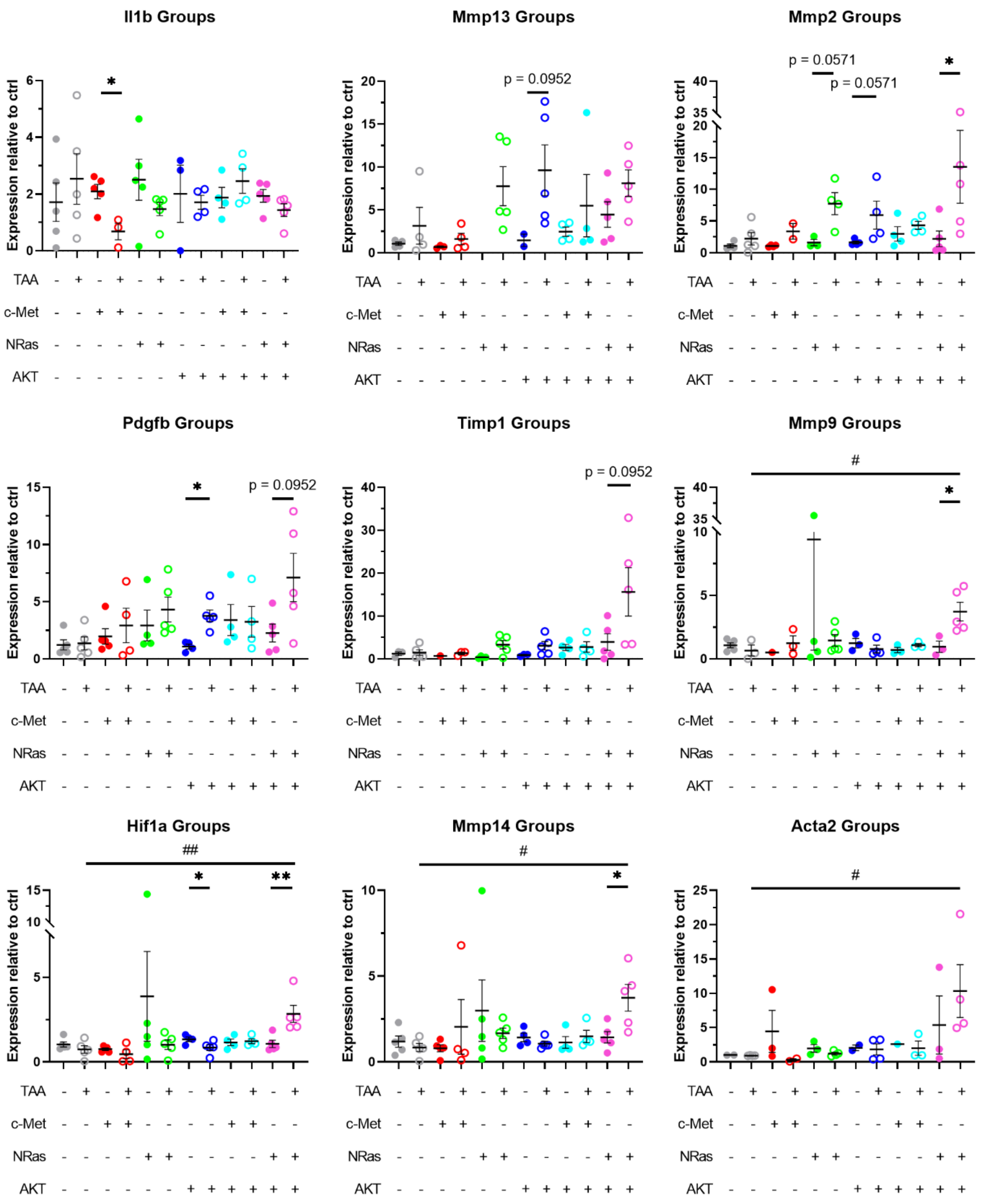

Supplementary Figure 10 (legend on next page)

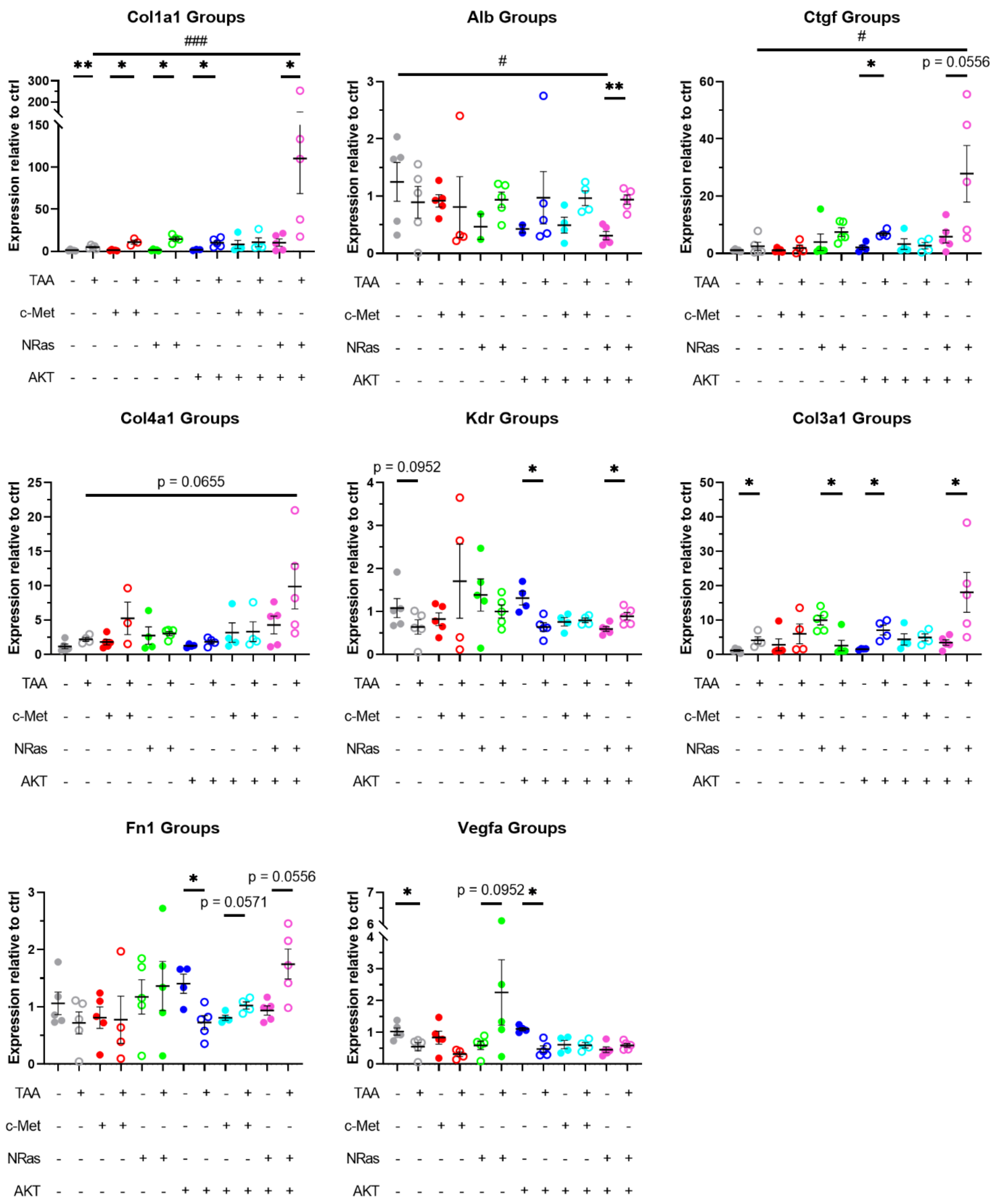

Supplementary Figure 10: Significantly altered genes, OpenArray fibrosis gene panel. Gene expression shown as fold-change relative to gene expression in SB + saline control group. Data are presented as mean ± SEM. TAA, thioacetamide; *$p < 0.05$, **$p < 0.01$ (Mann-Whitney test); $^{\#}p < 0.05$, $^{\#\#}p < 0.01$, $^{\#\#\#}p < 0.001$ (Dunn's post-hoc test).

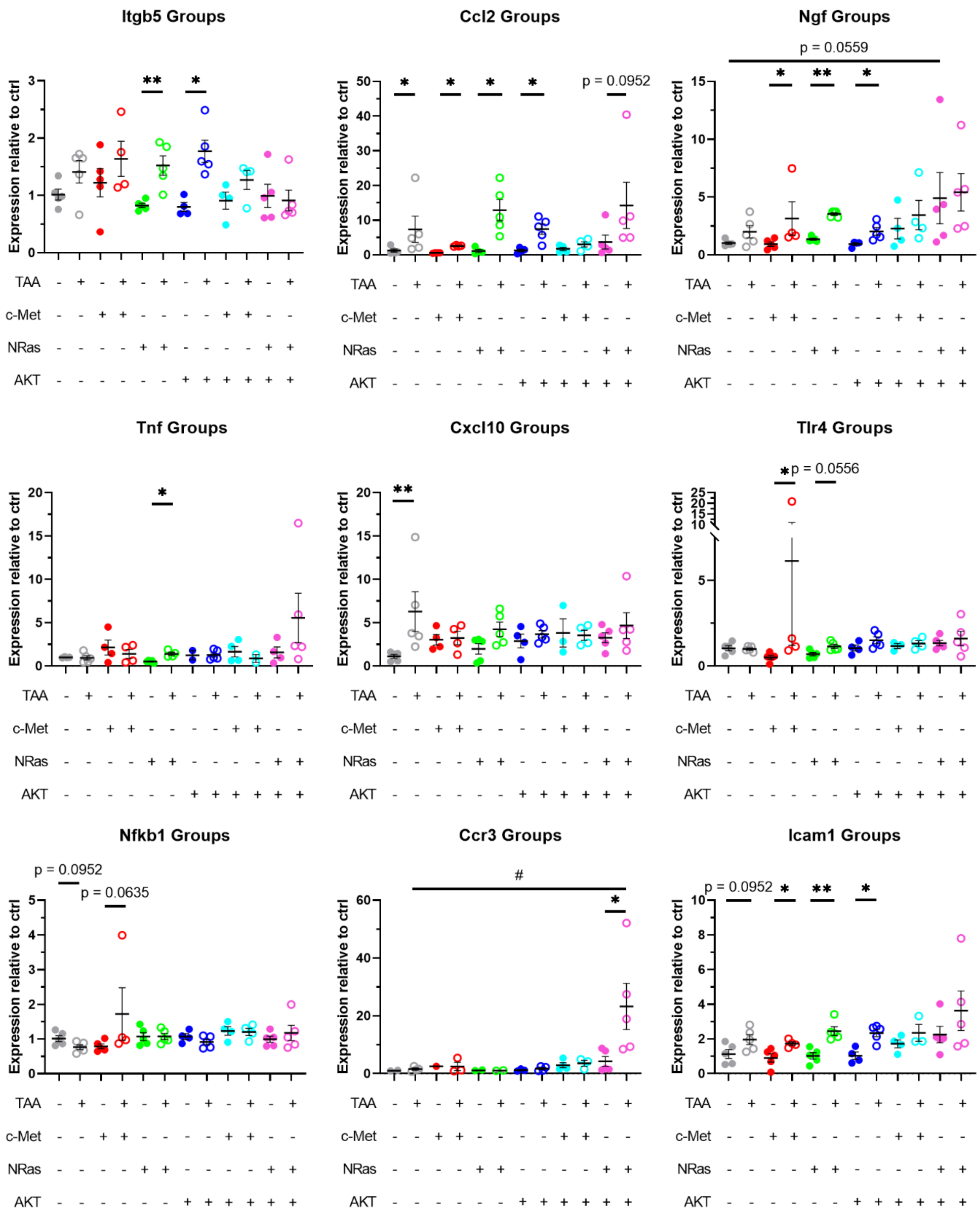

Supplementary Figure 11 (legend on next page)

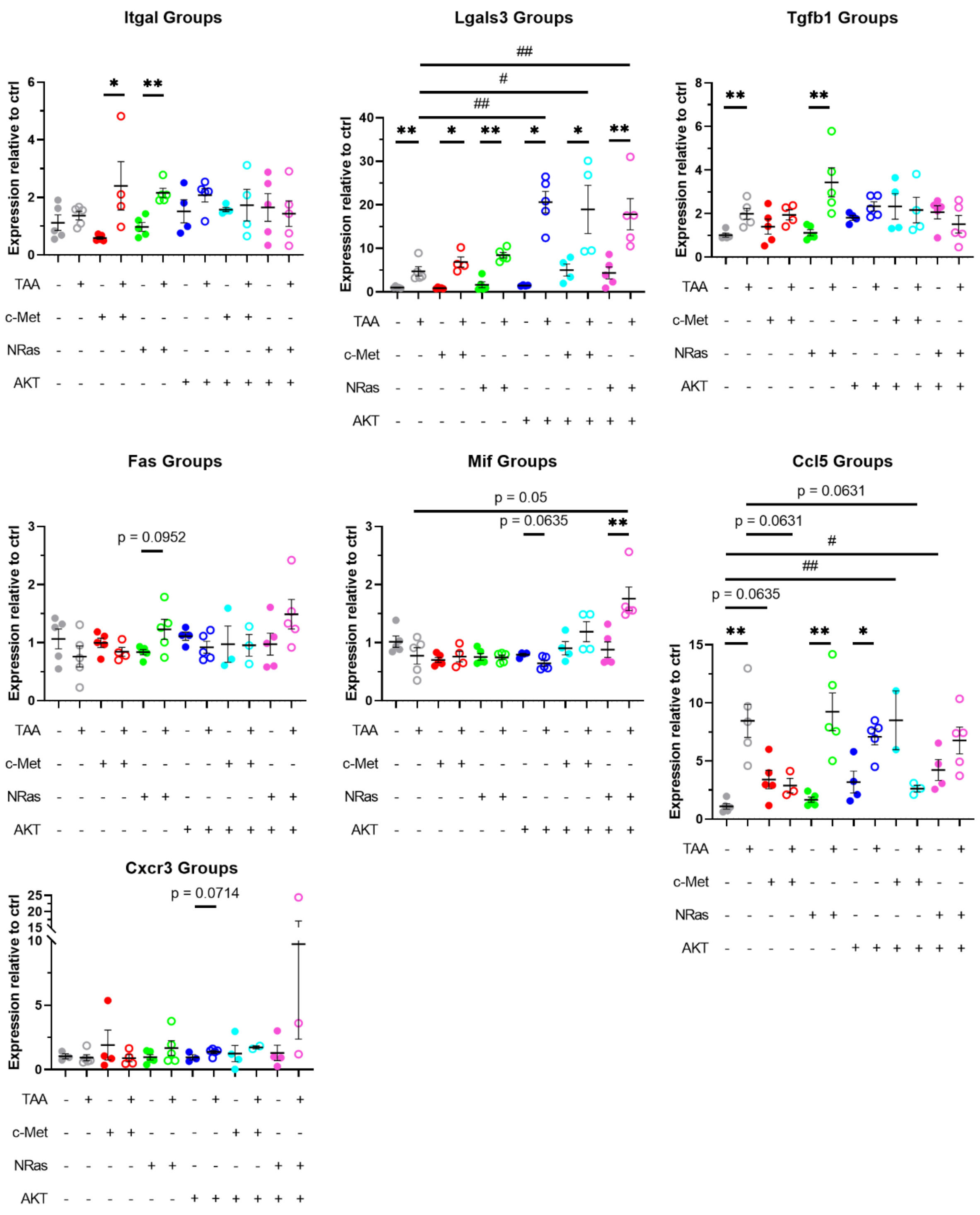

Supplementary Figure 11: Significantly altered genes, OpenArray immune gene panel. Gene expression shown as fold-change relative to gene expression in SB + saline control group. Data are presented as mean ± SEM. TAA, thioacetamide; $*p < 0.05$, $**p < 0.01$ (Mann-Whitney test); $^{\#}p < 0.05$, $^{\#\#}p < 0.01$ (Dunn's post-hoc test).

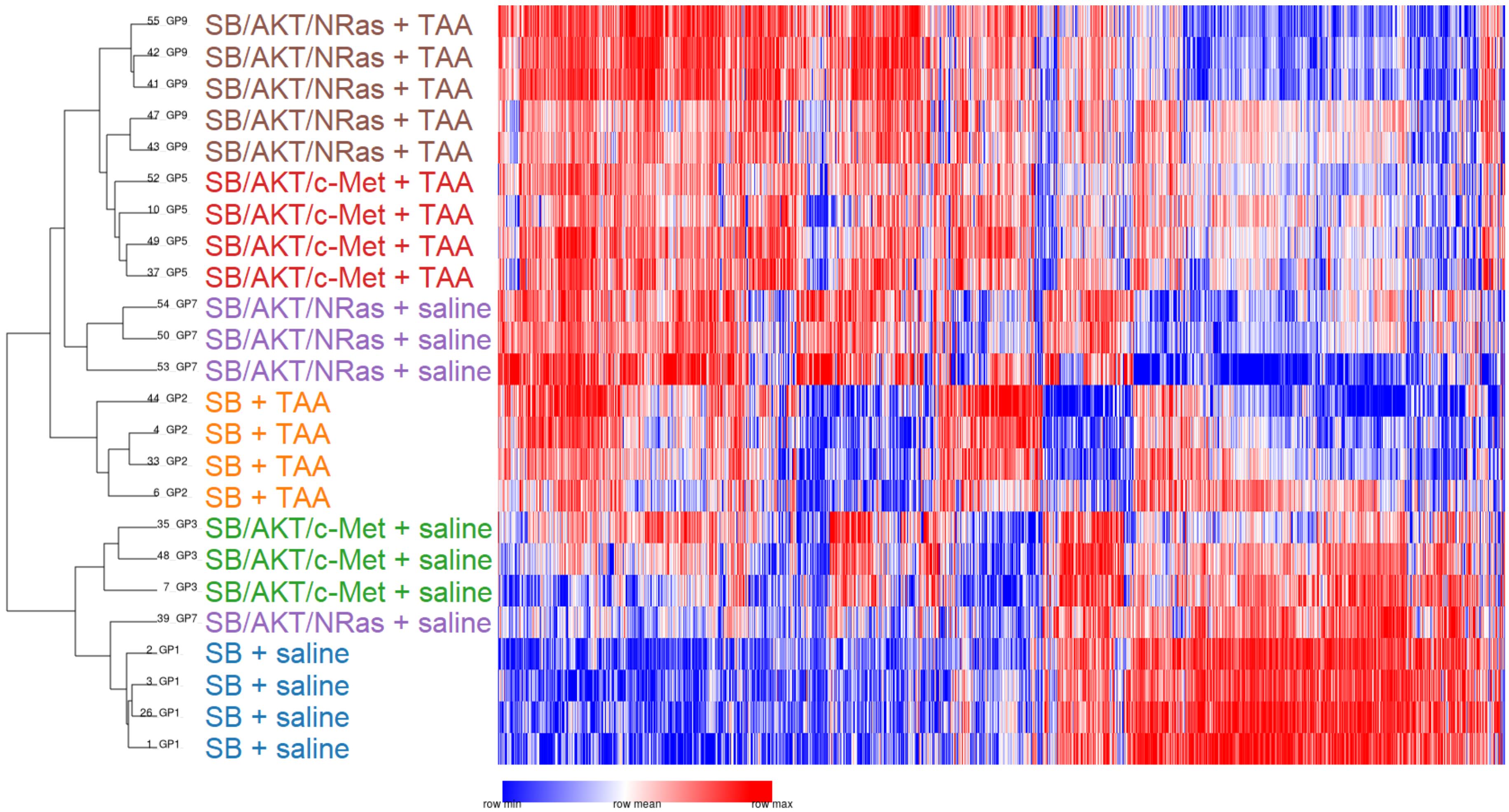

Supplementary Figure 12: Hierarchical clustering of samples by differentially expressed genes. Raw read counts were normalized using the VoomNormalize module (GenePattern), selected using multiple comparison false discovery rate < 0.05 filter and clustered using the HierarchicalClustering and HierarchicalClusteringViewer modules (GenePattern). For each gene (column), expression is shown on a blue-red colour gradient (lowest to highest) relative to the mean gene expression across samples (white).

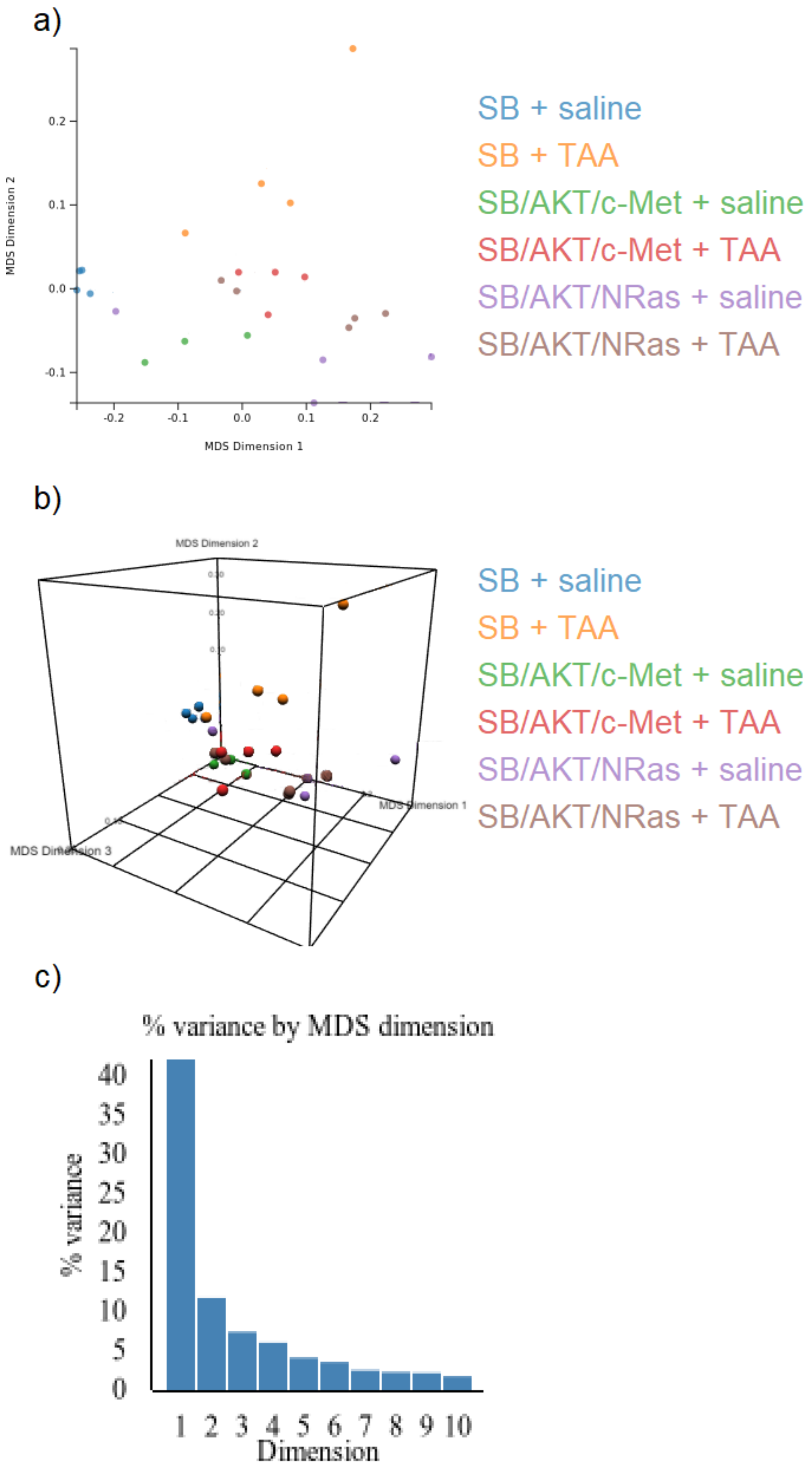

Supplementary Figure 13: Multidimensional scaling analysis of RNA-seq data. Plots were output from Degust. Sample labels were removed to improve clarity. (**a**) Multidimensional scaling (MDS) plot of MDS dimension 1 vs MDS dimension 2. (**b**) 3-dimensional MDS plot of MDS dimensions 1, 2, and 3. (**c**) Scree plot of MDS analysis.

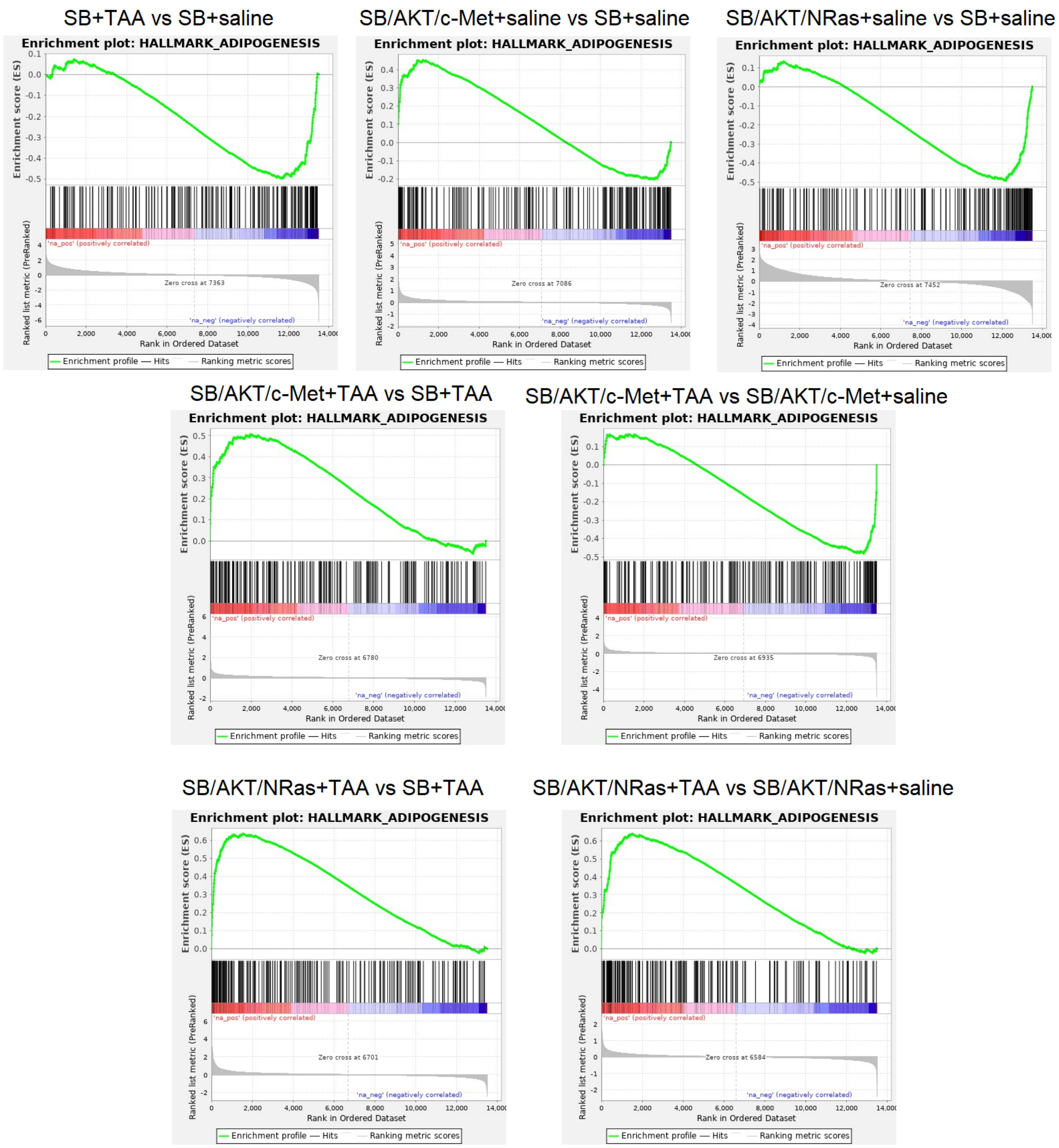

Supplementary Figure 14: GSEA enrichment plots of the Hallmark: Adipogenesis pathway.

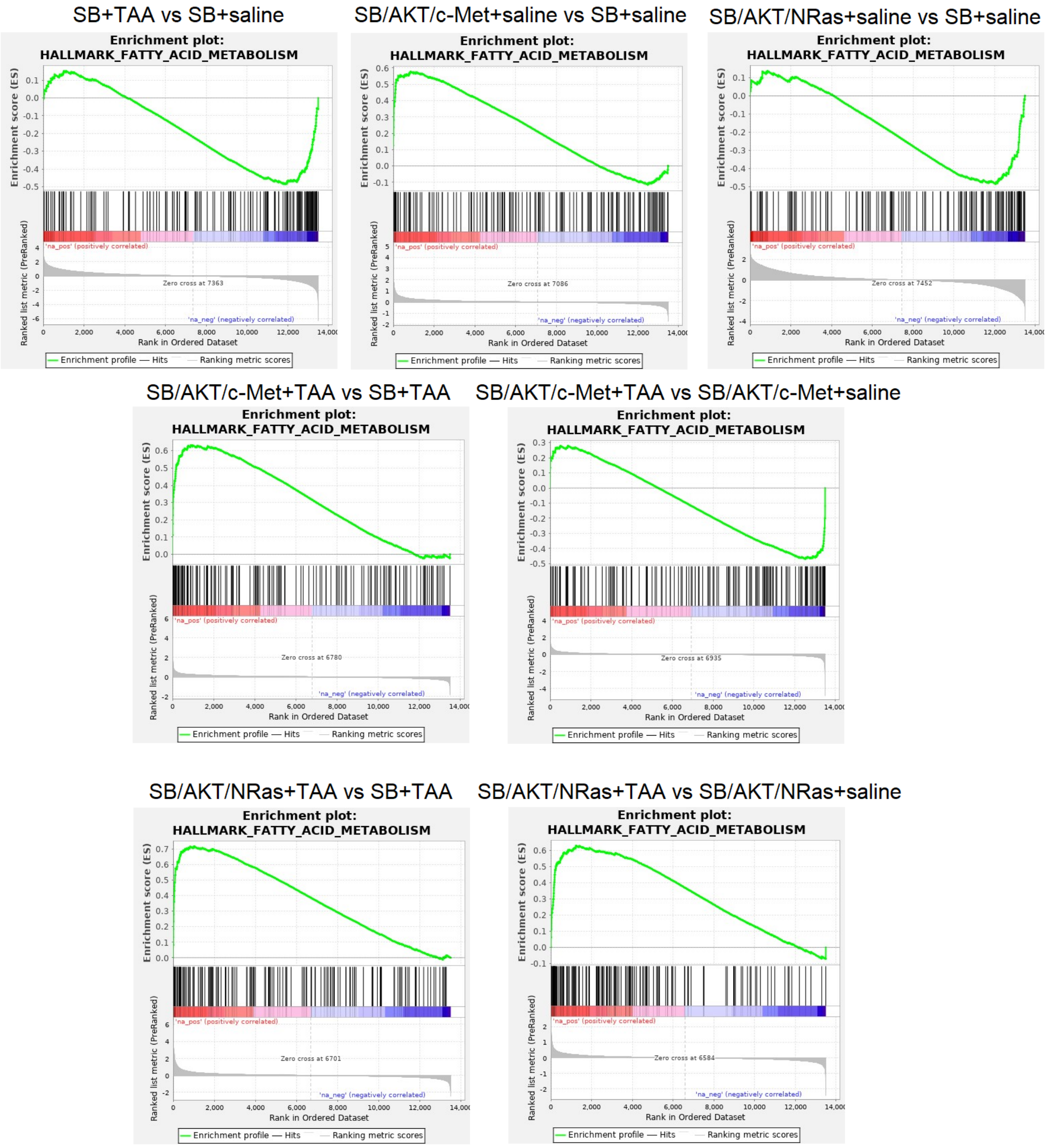

Supplementary Figure 15: GSEA enrichment plots of the Hallmark: Fatty acid metabolism pathway.
